# The role of spinal cord neuroanatomy in the variances of epidural spinal recordings

**DOI:** 10.1186/s42234-024-00149-2

**Published:** 2024-07-17

**Authors:** Danny V. Lam, Justin Chin, Meagan K. Brucker-Hahn, Megan Settell, Ben Romanauski, Nishant Verma, Aniruddha Upadhye, Ashlesha Deshmukh, Aaron Skubal, Yuichiro Nishiyama, Jian Hao, J. Luis Lujan, Simeng Zhang, Bruce Knudsen, Stephan Blanz, Scott F. Lempka, Kip A. Ludwig, Andrew J. Shoffstall, Hyun-Joo Park, Erika Ross Ellison, Mingming Zhang, Igor Lavrov

**Affiliations:** 1 Neural Lab, Abbott Neuromodulation, Plano, TX USA; 2 Department of Biomedical Engineering, Case Western Reserve University, Cleveland, OH USA; 3 Department of Veterans Affairs Medical Center, Advanced Platform Technology Center, Louis Stokes Cleveland, Cleveland, OH USA; 4 Department of Neurosurgery, Mayo Clinic, Rochester, MN USA; 5 Department of Biomedical Engineering, University of Wisconsin Madison, Madison, USA; 6 Wisconsin Institute for Translational Neuroengineering (WITNe), Madison, WI USA; 7 Department of Neurology, Mayo Clinic, Rochester, MN USA; 8 Department of Physiology and Biomedical Engineering, Mayo Clinic, Rochester, MN USA; 9 University of Wisconsin School of Medicine and Public Health, Madison, WI USA; 10 Department of Biomedical Engineering, University of Michigan, Ann Arbor, MI USA; 11 Biointerfaces Institute, University of Michigan, Ann Arbor, MI USA; 12 Department of Anesthesiology, University of Michigan, Ann Arbor, MI USA; 13 Department of Neurosurgery, University of Wisconsin-Madison, Madison, WI USA

**Keywords:** Spinal cord stimulation, Neuroanatomy, Epidural Spinal Recordings, Neuromodulation, Evoked Compound Action Potential, Spinally Evoked Motor Potentials

## Abstract

**Background:**

Spinal cord stimulation (SCS) has demonstrated multiple benefits in treating chronic pain and other clinical disorders related to sensorimotor dysfunctions. However, the underlying mechanisms are still not fully understood, including how electrode placement in relation to the spinal cord neuroanatomy influences epidural spinal recordings (ESRs). To characterize this relationship, this study utilized stimulation applied at various anatomical sections of the spinal column, including at levels of the intervertebral disc and regions correlating to the dorsal root entry zone.

**Method:**

Two electrode arrays were surgically implanted into the dorsal epidural space of the swine. The stimulation leads were positioned such that the caudal-most electrode contact was at the level of a thoracic intervertebral segment. Intraoperative cone beam computed tomography (CBCT) images were utilized to precisely determine the location of the epidural leads relative to the spinal column. High-resolution microCT imaging and 3D-model reconstructions of the explanted spinal cord illustrated precise positioning and dimensions of the epidural leads in relation to the surrounding neuroanatomy, including the spinal rootlets of the dorsal and ventral columns of the spinal cord. In a separate swine cohort, implanted epidural leads were used for SCS and recording evoked ESRs.

**Results:**

Reconstructed 3D-models of the swine spinal cord with epidural lead implants demonstrated considerable distinctions in the dimensions of a single electrode contact on a standard industry epidural stimulation lead compared to dorsal rootlets at the dorsal root entry zone (DREZ). At the intervertebral segment, it was observed that a single electrode contact may cover 20-25% of the DREZ if positioned laterally. Electrode contacts were estimated to be ~0.75 mm from the margins of the DREZ when placed at the midline. Furthermore, ventral rootlets were observed to travel in proximity and parallel to dorsal rootlets at this level prior to separation into their respective sides of the spinal cord. Cathodic stimulation at the level of the intervertebral disc, compared to an ‘off-disc’ stimulation (7 mm rostral), demonstrated considerable variations in the features of recorded ESRs, such as amplitude and shape, and evoked unintended motor activation at lower stimulation thresholds. This substantial change may be due to the influence of nearby ventral roots. To further illustrate the influence of rootlet activation vs. dorsal column activation, the stimulation lead was displaced laterally at ~2.88 mm from the midline, resulting in variances in both evoked compound action potential (ECAP) components and electromyography (EMG) components in ESRs at lower stimulation thresholds.

**Conclusion:**

The results of this study suggest that the ECAP and EMG components of recorded ESRs can vary depending on small differences in the location of the stimulating electrodes within the spinal anatomy, such as at the level of the intervertebral segment. Furthermore, the effects of sub-centimeter lateral displacement of the stimulation lead from the midline, leading to significant changes in electrophysiological metrics. The results of this pilot study reveal the importance of the small displacement of the electrodes that can cause significant changes to evoked responses SCS. These results may provide further valuable insights into the underlying mechanisms and assist in optimizing future SCS-related applications.

**Supplementary Information:**

The online version contains supplementary material available at 10.1186/s42234-024-00149-2.

## Introduction

Spinal cord stimulation (SCS) is an electrical neuromodulation technique that is commonly used to treat patients suffering from chronic pain who have not responded to the first-line standard of care treatment (Shealy et al. 1967; Melzack and Wall 1965; Moffitt et al. 2009). Although SCS has demonstrated successful clinical adoption, therapeutic outcomes continue to vary (Zhang et al. 2014), where the impact of the microanatomy surrounding the stimulation electrodes is poorly understood. The gate control theory suggests that inhibition of the pain signals along the dorsal column can be achieved by stimulation of large-diameter myelinated nerve fibers, primarily afferent Aβ-fibers (Melzack and Wall 1965). At the same time, variations in the clinical efficacy of SCS in managing pain are driven by multiple factors, including but not limited to epidural lead placement and location within the spinal column, lead migrations during postural changes or movement, and the functional state of the neuronal circuitry (Mekhail et al. 2022; Pahapill et al. 2020; Dombovy-Johnson et al. 2022).

Observing the evoked responses, such as evoked compound action potentials (ECAPs) and later responses, in epidural spinal recordings (ESRs) following SCS offers insight into the synchronous firing of nerve fibers in the spinal dorsal columns as well as SCS-induced muscle contractions near the stimulation site (Parker et al. 2012; Parker et al. 2013; Parker et al. 2020; Verma et al. 2023; Falowski, et al. 2022). Activation of the dorsal spinal columns has previously been linked to the orientation of electrical fields along the target fibers (Struijk et al. 1993; Rattay 1999; Rattay et al. 2000). Accordingly, the angles between the root fibers, spinal cord axis, and other anatomical parameters influence the electrophysiological outcome. Several computational and animal studies have demonstrated that electrode location and electrical field orientation are critical factors in the effectiveness of SCS (Coburn 1985; Struijk et al. 1993; Rattay 1999; Rattay et al. 2000). Recent studies have highlighted the importance of lead placement within the spinal segments and the role of spinal cord neuroanatomy in predicting the effectiveness of SCS-induced motor responses (Cuellar et al. 2017). Observed variations in segment-specific orientation of the spinal roots emphasized the importance of a targeted stimulation approach (Mendez et al. 2021). In a recent study, we explored how different referencing strategies influence various signal components in recorded ESRs, including amplitude and latency (Verma et al. 2023). We found that these variations may be useful in detecting lead migrations, a commonly observed side effect after lead placement (Mekhail et al. 2022; Dombovy-Johnson et al. 2022). In addition, dorsal rootlet activation may occur due to SCS-induced current leakage through the cerebrospinal fluid (CSF), activating the superficial dorsal rootlets entering the dorsal column (Capogrosso et al. 2013), which may be responsible for the electromyograph (EMG) component recorded in ESRs.

Dorsal root activation is considered the primary goal concerning the control of spinal reflexes and complex motor functions like locomotion (Lavrov et al., 2008; Capogrosso et al. 2013; Rowald et al. 2022). SCS modeling studies have highlighted several factors that influence dorsal root activation, including 1) spatial orientation and distance of rootlets to the stimulation contact, 2) vertebral segmentation and conductivity of surrounding tissue, 3) rootlet composition and dimension, including branching, curvature, and thickness (Solmaz et al. 2015; Mendez et al. 2021), and 4) electrical stimulation parameters and electrode configurations (Struijk et al. 1993; Holsheimer et al. 1995; Holsheimer et al. 1997; Holsheimer 1998; Barolat 1998; Manola et al. 2005; Capogrosso et al. 2013; Anaya et al. 2020; Rogers et al. 2022). However, the dorsal root fibers’ contribution and orientation to SCS-evoked ECAP and EMG components in ESRs remains unclear. Building on these findings, this pilot study assessed how minimal variations in stimulation location relative to the local spinal cord anatomy could impact ESRs during SCS.

## Methods and materials

### Experimental subjects and surgical procedures

**Fig. 1:**
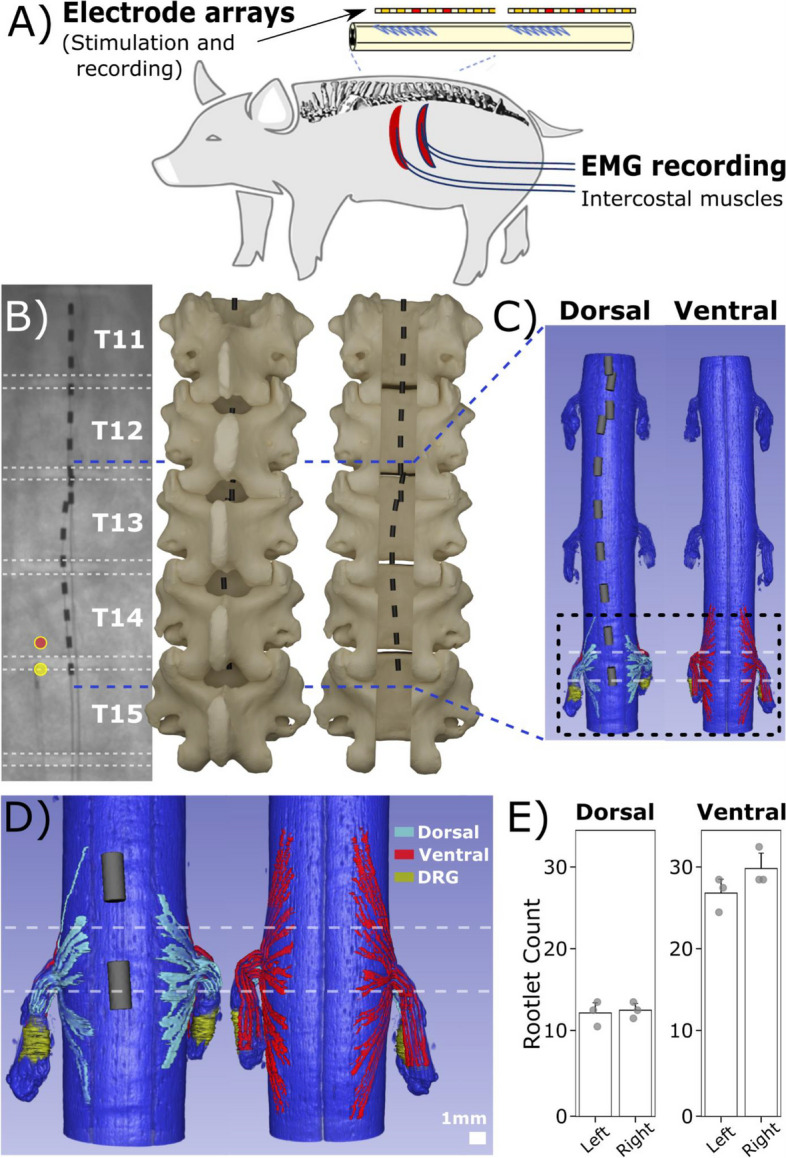
3D-model reconstruction of implanted epidural leads and spinal cord. **A** Experimental diagram. **B** For modeling purposes, x-ray images were taken from a representative subject’s (S0) thoracic vertebra segments with implanted epidural Octrode™ leads. Circular markers indicate the pair of electrode contacts used for SCS. Dashed white horizontal lines indicate intervertebral discs based on x-ray projections. 3D-models of the vertebra were generated to illustrate epidural lead placements with respect to the spinal column. **C** The subject’s spinal cord was reconstructed using microCT at levels around T12-T14 with epidural leads overlaid at ventral and dorsal viewing planes. **D** Reconstructed microCT segment of the spinal cord at T14 shows segmented dorsal and ventral rootlets and DRG. **E** Bilateral comparison of both dorsal and ventral rootlet counts (T12-T14) showed no significant differences in spinal rootlet count

A domestic male 10 weeks old swine (S0; 30 kg) was used for computed tomography (CT) imaging and microdissection of the spinal cord. In a separate cohort, four domestic swine both sexes 8-12 weeks old (S1, S2, S3, S4; 27-46 kg) were used for SCS and electrophysiology recording (Fig. 1A). Study procedures with regards to CT imaging were approved by the University of Wisconsin-Madison’s Institutional Animal Care and Use Committee, and study procedures with regards to recording, were conducted with the approval of the Mayo Clinic Institutional Animal Care and Use Committee, and in accordance with the National Institutes of Health Guidelines for Animal Research (Guide for the Care and Use of Laboratory Animals). Animals were kept in separate cages in a controlled environment (constant temperature at 21 °C and humidity at 45%) on a 12-hour light/dark cycle with ad libitum access to water and were fed once daily. The surgical approach has been described in detail previously (Cuellar et al. 2017; Verma et al. 2023). Intramuscular telazol (6 mg/kg), xylazine (2 mg/kg), and glycopyrrolate (0.006 mg/kg) were administered for anesthesia induction. An intramuscular injection of buprenorphine was given as an analgesic (0.03 mg/kg). Fentanyl was continuously administered during surgery (2-5 mg/kg/h) as an analgesic. Subjects were endotracheally intubated and maintained at 1.5–3% isoflurane throughout the surgery.

## Cone Beam Computed Tomography (CBCT) imaging for 3D-modeling of the spine

CBCT images were collected prior to and after surgical implantation of the epidural leads. In brief, a biplane C-arm system (Axion Artis, Siemens Healthineers, Forchheim, Germany) was used to image the subject (S0) in a prone position. Images were obtained with the following parameters: 12 second rotation, 0.47 mm spatial resolution (isotropic), and 24 x 24 x 17 cm^3^ field-of-view. After initial imaging of the swine, a laminectomy was performed to expose the spinal processes through cautery and blunt dissection. Spinal processes were removed, and windows were created between each level of the spinal cord from L3-T15. Epidural leads containing eight platinum-iridium contacts (Octrode^TM^, Abbott, Plano, TX) were implanted above the dura in the epidural space using fluoroscopic guidance (Fig. 1B). The caudal-most contact was positioned around the T14-T15 intervertebral disc plane. Dimensions of individual electrode contacts were 1.3 mm diameter, 3 mm contact length, and 4 mm inter-contact spacing. Afterward, a final CBCT image was collected of the spine with implanted epidural leads.

### Spinal cord tissue extraction and processing

Following euthanasia, a microdissection was performed on one subject (S0) to expose the intact dura and spinal cord and then remove the spinal cord from T9-L5. Care was taken to excise the dorsal root ganglion (DRG) and spinal roots on both the left and right sides of the cord. Histology marking dye (Davidson Marking Systems, Minneapolis, MN) was placed at locations of interest, such as at the level of stimulation and on the dorsal aspect of the roots - to maintain orientation. Spinal cord roots were transected lateral to the DRG where possible, approximately 3-5 cm from the spinal cord, and the spinal cord was removed and stored in 10% neutral buffered formalin for 5-7 days.

### 3D-model generation of thoracic vertebrae with implanted epidural leads

Post-operative CBCT images containing epidural lead implants were segmented to obtain the contact positions relative to vertebrae. Pre-operative CBCT images were segmented to generate 3D surfaces of the subject’s vertebral column (T10-T15) and aligned to respective vertebrae from post-operative CBCT images. 3D surfaces were exported into Blender (Blender Foundation, Netherlands) to visualize the location of the implanted epidural leads with respect to the spinal column (Fig. 1B).

### MicroCT imaging and image reconstruction of extracted spinal cord

The subject’s (S0) dissected spinal cord was incubated on a nutating shaker for five days in 1% osmium tetroxide (OsO_4_) prepared with deionized water (DIW). The sample was dehydrated through incubation in diluted ethanol solution with DIW for 30 minutes, followed by three incubation cycles of 70% ethanol and 95% ethanol, respectively. The sample was kept in 70% ethanol for long-term storage prior to microCT imaging (Quantum GX2 microCT System, Perkin Elmer, Waltham, MA). The sample was placed onto a 36 mm bed and imaged with x-ray parameters set at 90 keV and 80 µA with aluminum and copper filters. The field-of-view was set at 36 mm and spanned a length of 1.8 cm along the sample. The sample was imaged with 0.3 cm overlap between each adjacent scan for post-image stitching. Final pre-reconstruction resolution for images was set at 72 µm voxel followed with post-hoc sub-block reconstruction at 36 µm resolution, resulting in dimensions of 18.4 x 18.4 x 18.4 mm (Database V3.5.3.110, Perkin Elmer, Waltham, MA). The reconstructions were then stitched together via pairwise stitching and exported as a .tiff stack. The .tiff stack was imported into 3D Slicer using the SlicerMorph plugin (Fedorov et al. 2012). Additionally, a user-written Python script was used to locate the centroids of the electrode contacts based on post-operative CBCT scans. Imaging artifacts were removed, and electrode contacts were reconstructed and exported for image overlay against the spinal cord. Final image reconstruction of the spinal cord with epidural lead overlay was accomplished with 3D Slicer (www.slicer.org). In brief, the dimensions of microCT-generated images of the DRG were compared to the dimensions of the CBCT-generated images of the interforamen. Post-fixation tissue shrinkage was estimated to be ~23%. 3D volume renderings were scaled and adjusted accordingly.

### Spinal cord stimulation

In four subjects (S1, S2, S3, S4), epidural leads were implanted onto the dorsal columns of the spinal cord (Cuellar et al. 2017; Verma et al. 2023). Stimulation artifact was minimized by using an asymmetric, charge-balanced waveform with an anodic-leading rectangular pulse with a duration of 400μs, followed by a cathodic pulse with a duration of 80μs. The second phase of the waveform had an amplitude five times greater than the amplitude of the leading phase (Grill and Mortimer 1995). Stimulation amplitudes reported in this study were defined using the second phase of the stimulation waveform. A pair of electrode contacts positioned at the caudal most end of the stimulation lead was utilized to evoke epidural spinal activity. The caudal-most contact was intentionally positioned around the T11-T12 intervertebral disc plane. Cathodic stimulation at this level was referred to as ‘on-disc’ stimulation, where the effective second phase stimulation was located on or near the intervertebral disc plane. Cathodic polarity for the pair of stimulation contacts was flipped to evaluate electrophysiology changes in relation to stimulating the intersegments of the spine. Changes in electrophysiology were evaluated in recorded ESRs and intramuscular electromyography (EMG) recordings. Furthermore, the stimulation lead was manually displaced laterally from midline of the dorsal columns to simulate lateral lead migration and evaluate its effects on electrophysiology recordings. The stimulation waveform was delivered at 38 Hz to represent therapeutic applications of SCS and avoid 60 Hz harmonics. The stimulation was delivered with the battery-isolated Subject Interface Module (Tucker Davis Technologies, Inc., Alachua, FL). Epidural leads were connected to a TDT SBOX16 (Tucker Davis Technologies, Inc., Alachua, FL) via a trial stimulation adapter, Medusa cable (Threshold Neurodiagnostics, Minoa, NY).

### ESR and intramuscular EMG recordings

We performed *in vivo* electrophysiological experiments to record the ESR from the spinal cord and spinal-evoked intramuscular EMG from intercostal muscles during SCS. To ensure secure placement of epidural leads, minimal laminectomies were performed on L1 and the five rostral thoracic segments. This allowed for the passage of the epidural leads into the epidural space and provided a means to verify their precise positioning. Using fluoroscopic guidance, fine adjustments of the epidural leads in relation to the dorsal spinal cord anatomy were performed. During the laminectomy, connective and fat tissue were removed while keeping the dura mater intact. ESRs were recorded concurrently on all 14 non-stimulation electrode contacts along the two implanted epidural leads, with two designated stimulation contacts (Fig. 1B). For intramuscular EMG recording, a pair of two stainless-steel needle electrodes were placed intramuscularly into the two lowest intercostal muscles. A stainless-steel wire (AS631, Cooner Wire, Chatsworth, CA) served as the reference electrode and was inserted into the paravertebral muscles, lateral of the surgical site, at a similar level to the most rostral electrode contact. Additional tools for electrophysiology recording included the TDT WS4 Computer, RZ5D Bioamp Processor, IZ10 Stimulation/amplifier, and Synapse software (Tucker Davis Technologies, Inc., Alachua, FL). Electrophysiology recordings were digitized at a sampling rate of ~25 kHz and were processed offline through band-pass filtering (100 Hz high-pass, first-order Butterworth and 3 kHz low-pass, Gaussian) in Python 3.7 using the SciPy package.

### Data analysis and quantifications

The pyeCAP package (https://pypi.org/project/pyeCAP/) was used in Python 3.7 for offline handling and analysis of electrophysiology data. Recorded waveforms were analyzed and compared at current thresholds for ESRs. Signal quantifications for recorded ESRs were conducted for individual components representing ECAP (from the end of the stimulation artifact to 2 ms following) and EMG (2-10 ms following the end of the stimulation artifact). Additionally, intramuscular EMG recordings were quantified within a similar time window (2-10 ms after the end of the stimulation artifact). In brief, the root-mean-square (RMS) was computed within established time windows for respective ESR components and intramuscular EMG recordings. Quantifications in this study were presented as mean ± standard error (SEM). Paired t-tests were performed to evaluate significant differences in calculated RMS values for ESRs and intramuscular EMG recordings when comparing 1) ‘on-disc’ and ‘off-disc’ stimulations and 2) SCS at medial and lateral positions of the epidural lead. Conduction velocity was estimated using a linear regression between the recording-stimulating contact distance and ECAP latency (indicated as the time that the largest negative peak was detected for a set time window with respect to the recording channel of interest).

## Results

### Visualization of dorsal and ventral rootlets in relations to implanted epidural leads

Electrode arrays were implanted into the epidural space of the spinal cord (Fig. 1A). High-resolution imaging using microCT was performed for a swine spinal cord from subject S0. X-ray imaging provided an initial overview of the implanted epidural leads with respect to vertebrae of the spine (Fig. 1B). 3D-models of the spine containing the epidural leads were generated based on pre- and post-operative CBCT images (Fig. 1B). Pre-operative CBCT images were used to generate the surfaces of individual vertebrae. Post-operative CBCT images were used to obtain positions of individual electrode contacts with respect to the surrounding vertebrae. An illustrative laminectomy was provided to demonstrate epidural lead positioning along the spinal column (Fig. 1B). The spinal cord was processed and stained for microCT imaging to visualize the micro-neuroanatomy (Fig. 1C). To visualize the location of the epidural leads in relation to the neuroanatomy, 3D-volume renderings of the spinal cord were generated and aligned with post-operative CBCT images. High-resolution imaging further provides visualization and distinct features of the DRG and DREZ surface area coverage (Fig. 1D). The lateral plane of the reconstructed spinal column reveals a close spatial relationship between the dorsal and ventral rootlets to one another, with individual rootlets having diameters as small as 150 µm (Supplementary Fig. 2). From T12-T14, the number of dorsal and ventral rootlets on both lateral columns of the spinal cord was comparable. Also, there were higher counts of spinal rootlets located on the ventral plane of the spinal cord compared to the dorsal column (Fig. 1E). The DREZ length of the left side of T14 was measured at 15.3 mm while the DREZ length of the right side measured at 11.2 mm. These values fit within the range, from about 10 to 20 mm, outlined in a similar study where measurements were manually performed on lumbosacral spinal levels (Cuellar et al. 2017). In addition, the dorsal rootlet counts, and the rostral/caudal angles were similar to T15/L1 measurements found in Cuellar et al. (15.1 ± 6.49 mm, Cuellar et al. 2017).

**Fig. 2:**
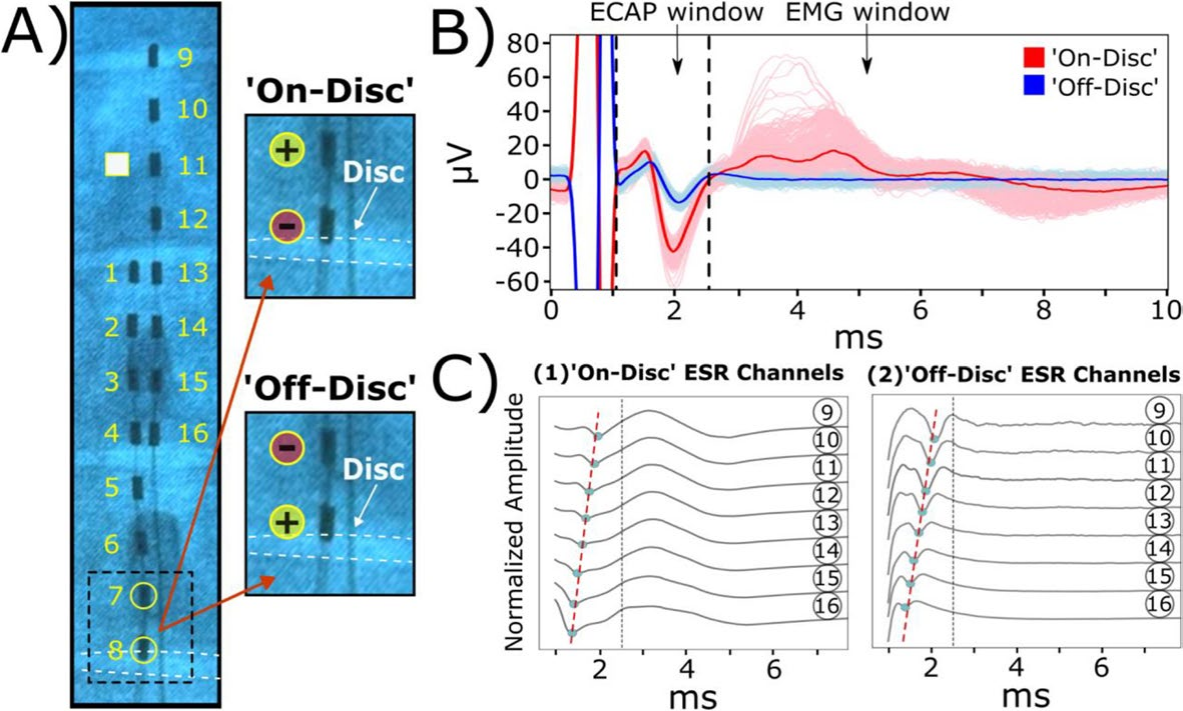
Evoked ESRs are responsive to the anatomical location of the stimulating cathode. Representative data from subject S1. **A** Diagram of implanted epidural leads with the stimulating cathode located either on or off the intervertebral disc. Contact 11 (white square) on the second lead indicates the recording channel used to showcase ESR waveforms. Circular markers on the epidural stimulation lead indicate electrode contacts as either anode (yellow) or cathode (red). Dashed white horizontal lines indicate intervertebral disc based on x-ray projections. **B** ESR waveforms were represented as 300 individual traces with a median trace overlay at a stimulation amplitude of 4.0 mA. We further defined the ECAP window (1-2.5 ms) and EMG window (3-13 ms) for recorded ESRs. **C** Recorded ESRs from 8/8 contacts of the recording epidural lead were plotted to evaluate conduction velocities. The contacts located on epidural leads were ~7 mm apart from center-to-center. Large stimulation artifacts were observed in other recording channels (electrode contacts 1-6) located near the pair of stimulating electrode contacts. Cathodic ‘on-disc’ stimulation generated distorted signals within the ECAP window, with an estimated conduction velocity of 75.8 m/s, including visible EMG signals afterward (C1). When switched to the ‘off-disc’ stimulation, a more distinct waveform was recorded in the ECAP window, where conduction velocity was estimated to be at 70.9 m/s, with no observable signals in the EMG window (C2). Dashed line around 2.5 ms indicates the separation between the ECAP and EMG windows

### Cathodic stimulation at the intervertebral segment substantially influences evoked ESRs

Dorsal and ventral rootlets were observed to enter the spinal cord primarily at the intervertebral segment (Fig. 1C, 1D). To characterize SCS-induced ESRs in relation to the intervertebral segment, the caudal-most electrode of the stimulation lead (contact 8) was positioned at the level of the intervertebral disc. Cathodic stimulation, or ‘on-disc’ stimulation, was performed at or near the disc level (Fig. 2A). Stimulation polarities were flipped for contact 7 and contact 8 (7 mm inter-electrode distance rostrally) where the cathode was located away from the intervertebral disc level, labeled as ‘off-disc’ stimulation. One subject (S1) was used to compare cathodic stimulation for ‘on-disc’ and ‘off-disc’ stimulation at 4.0 mA, and clear evoked signals in the ECAP and EMG windows were observed (Fig. 2B). RMS value was calculated for ECAP and EMG windows of recorded ESRs in evaluating changes in evoked responses for ‘off-disc’ and ‘on-disc’ stimulations. ‘Off-disc’ stimulation produced significantly weaker responses (-89.8 ± 0.16%) within the ECAP window (*p*<0.005, paired t-test; Fig. 2B, 3A1). However, the evoked waveforms during ‘on-disc’ stimulation included a significant motor response leading to a larger late response within the EMG window (Fig. 2B). In addition, significantly weaker responses were evoked (-96.0 ± 0.09%) within the EMG window during ‘off-disc’ stimulation (*p*<0.005, paired t-test) (Fig. 2B, 3A2). The latency and morphology of the recordings were tracked throughout the recordings (Fig. 2C). ‘On-disc’ stimulation evoked waveforms within the ECAP window (up to 2 ms following the end of the stimulation artifact) throughout the recording lead, followed by larger evoked responses (EMG component) occurring within the time window of 1.5-4.5 ms (Fig. 2B, 2C left panel). Conduction velocity was estimated by measuring slope of the signal latency in ECAP window (tracking the largest negative peak within a given time window). For subject S1, ‘on-disc’ stimulation produced an estimated conduction velocity of 75.8 m/s at a stimulation amplitude of 4.0 mA, and ‘off-disc’ produced an estimated conduction velocity of 70.9 m/s (Fig. 2C).

**Fig. 3:**
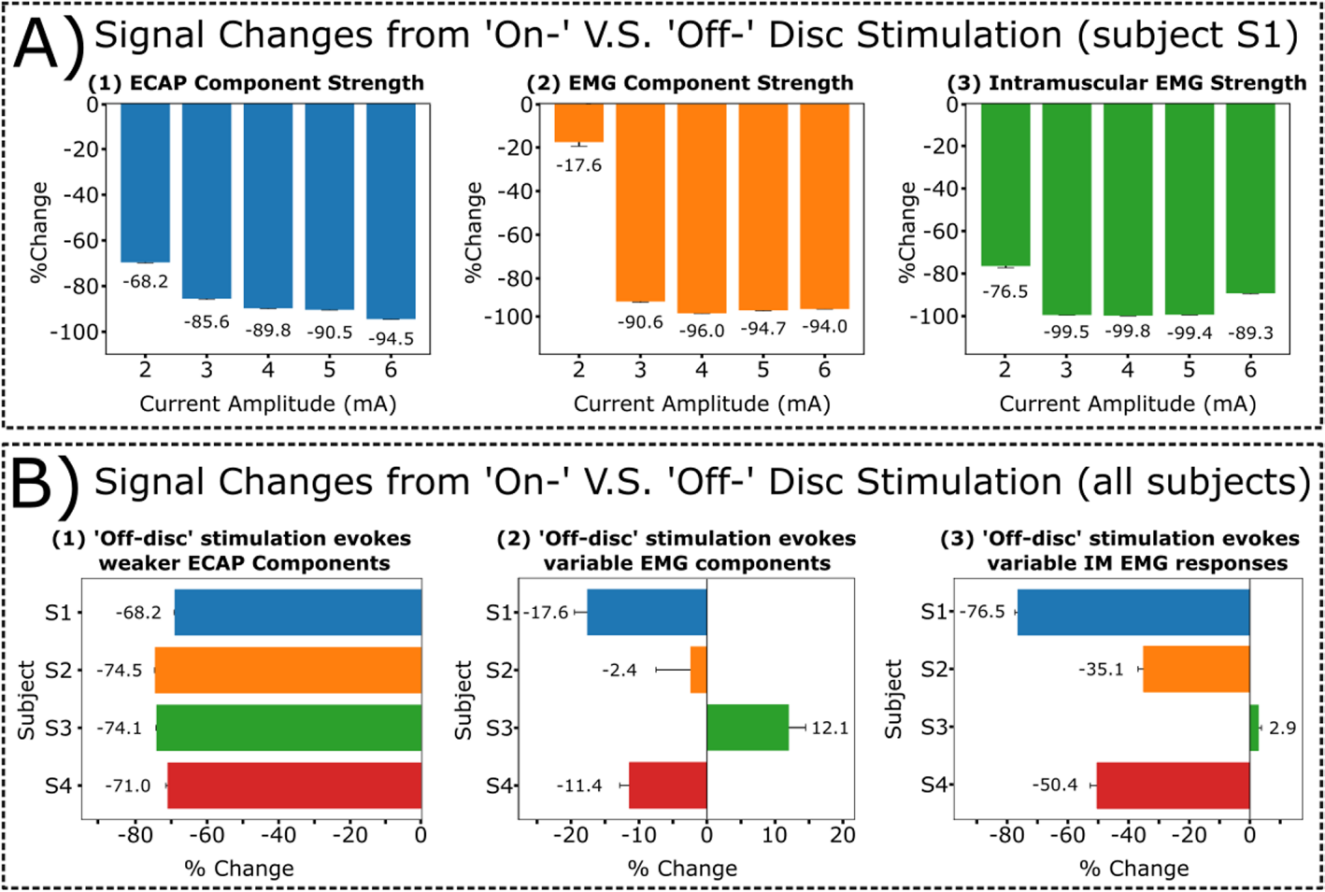
Quantification for recorded ESR components and intramuscular EMG recordings when comparing ‘on-disc’ stimulation to ‘off-disc’ stimulation. Motor thresholds for ‘on-disc’ stimulation of subjects (*n*=4) were verified within intramuscular EMG recordings and as follows: S1, 2.0 mA; S2, 6.0 mA; S3, 2.0 mA; S4, 2.9 mA). **A** Data from respective subject S1 shows signal strength for collected ESRs changed from ‘on-disc’ to ‘off-disc’ stimulation, shown as (1) ECAP (2.0 mA: -68.2 ± 0.61%; 3.0 mA: -85.6 ± 0.30%; 4.0 mA: -89.8 ± 0.16%; 5.0 mA: -90.5 ± 0.15%; 6.0 mA: -94.5 ± 0.07%) and (2) EMG components (2.0 mA: -17.6 ± 1.86%; 3.0 mA: -90.6 ± 0.31%; 4.0 mA: -96.0 ± .09%; 5.0 mA: -94.7 ± 0.13%; 6.0 mA: -94.1 ± 0.08%), and (3) intramuscular EMG (IM) recordings (2.0 mA: -76.5 ± 0.81%; 3.0 mA: -99.5 ± 0.01%; 4.0 mA: -99.8 ± 0.01%; 5.0 mA: -99.4 ± 0.03%; 6.0 mA: -89.3 ± 0.24) from intercostal muscles were significantly weaker across various stimulation amplitudes. **B**. Changes in signal strength from ‘on-disc’ to ‘off-disc’ stimulation were observed at motor thresholds for all subjects (*n*=4) under evoked motor thresholds within recorded ESRs, including (1) ECAP (S1: -68.2 ± 0.61%; S2: -74.5 ± 0.1%; S3: -74.1 ± 0.1%; S4: -71.0 ± 0.43%) and (2) EMG components (S1: -17.6 ± 1.86%; S2: -2.40 ± 5.07%; S3: 12.1 ± 2.46%; S4: -11.4 ± 1.40%) and (3) intramuscular EMG (IM) recordings (S1: -76.5 ± 0.81%; S2: -35.1 ± 1.76%; S3: 2.90 ± 0.91%; S4: -50.4 ± 2.23%). Additional plots for subject comparisons containing RMS values are provided in Supplementary Fig. 8

Next, subject S1’s evoked responses in ESRs and intramuscular EMG recordings were evaluated for ‘on-disc’ and ‘off-disc’ stimulation at various stimulation amplitudes from a range of 2.0-6.0 mA (motor threshold at ‘on-disc’ stimulation: 2.0 mA; Fig. 3A). RMS was calculated for ECAP and EMG components of recorded ESRs and intramuscular EMG (IM, time window: 3-13 ms) recordings. ‘Off-disc’ stimulation produced significantly weaker responses within the ECAP window for all observed stimulation amplitudes compared to ‘on-disc’ stimulation (*p*<0.005, paired t-test; Fig. 3A1). Furthermore, significantly weaker motor activity was evoked during ‘off-disc’ stimulation across all investigated stimulation amplitudes for subject S1, as observed in EMG component of recorded ESRs and intramuscular EMG recordings (*p*<0.005, paired t-test; Fig. 3A2, 3A3).

To evaluate if this trend was consistent, changes in evoked responses from ‘on-disc’ to ‘off-disc’ stimulation were measured for all tested subjects (*n*=4) at their respective motor thresholds during ‘on-disc’ stimulation (Fig. 3B). At motor threshold, ‘off-disc’ stimulation consistently produced significantly weaker responses within the ECAP window for all subjects compared to respective ‘on-disc’ stimulation responses (*p*<0.005, paired t-test; Fig. 3B1). Evoked responses within the EMG window of recorded ESRs and intramuscular EMG recordings were not consistent when comparing results across different subjects. Signal strengths for EMG components were weaker for ‘off-disc’ stimulation for subjects S1 and S4 (*p*<0.005, paired t-test; Fig. 3B2). Subject S2 had a weaker response during ‘off-disc’ stimulation. However, there were little variances introduced (-2.40 ± 5.07%). For subject S3, ‘off-disc’ stimulation evoked significantly larger responses within the EMG component compared to the rest of the cohort (*p*<0.005, paired t-test; Fig. 3B2). This relationship was also observed in the intramuscular EMG recordings where ‘off-disc’ stimulation evoked significantly weaker motor activity in subjects S1, S2, and S4 but not subject S3 (*p*<0.005, paired t-test; Fig. 3B3). Further data from subjects S2 and S3 were analyzed to evaluate the cause of the variable responses.

**Fig. 4:**
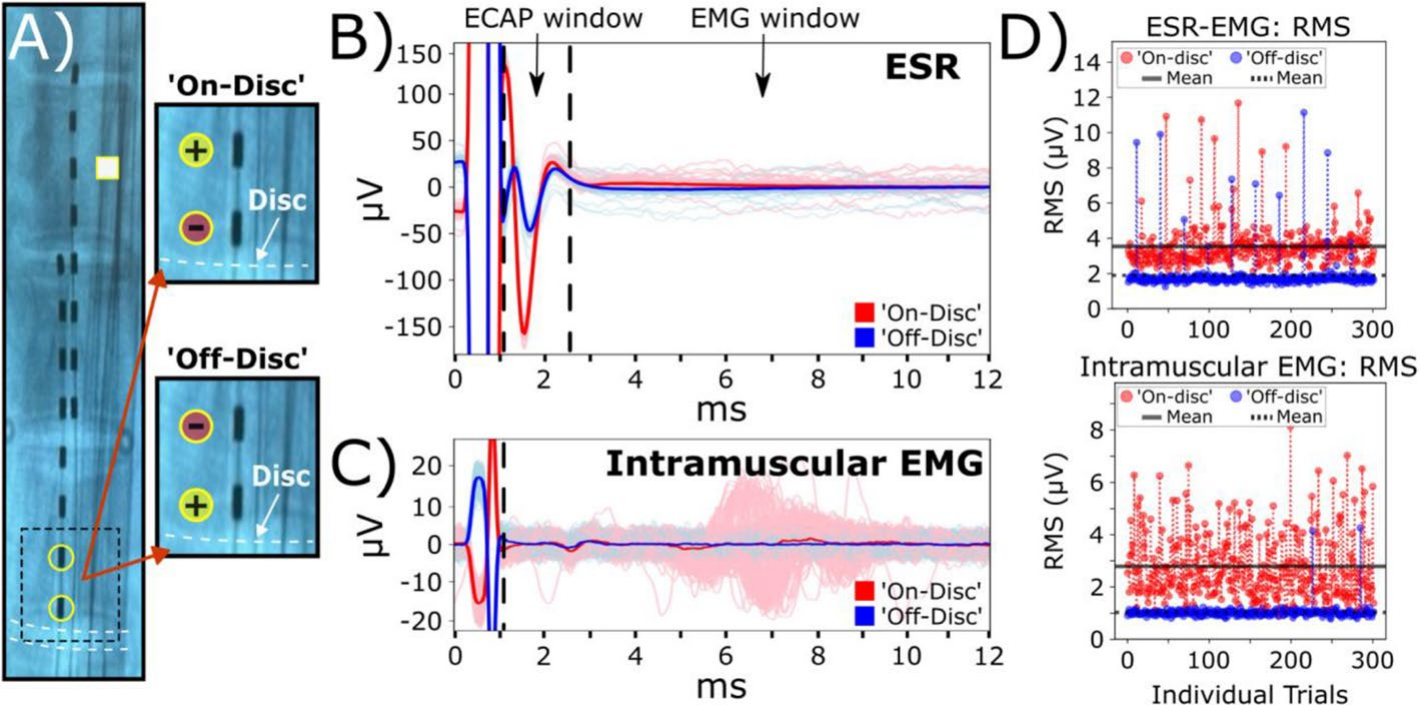
Stimulation contacts located adjacent to the intervertebral disc rather than beneath showed no observable motor response within recorded ESRs. Representative data from subject S2 with stimulation amplitude of 6.0 mA. **A** Diagram of implanted epidural leads with the stimulating cathode located as either on/near or off the intervertebral disc. Contact 11 (white square) on the second lead indicates the recording channel for reported ESR waveforms. Circular markers on the epidural stimulation lead indicate electrode contacts as either anode (yellow) or cathode (red). **B** Recorded ESRs with ECAP (1-2.5 ms) and EMG windows (3-13 ms) and **C** intramuscular EMG (IM) recordings were represented as 300 individual traces with a median trace overlay. **D** Calculated RMS for individual trials of the EMG components of recorded ESRs (top) and intramuscular EMG recordings (bottom) were plotted with respect to ‘on-disc’ (red) and ‘off-disc’ (blue) stimulations. Mean RMS values were represented as solid lines (‘on-disc’) and dashed lines (‘off-disc’)

Upon further inspection, subject S2’s caudal-most electrode contact was positioned above the boundary of the intervertebral disc, ~4 mm rostral from the intervertebral disc along the rostro-caudal axis (Fig. 4A) when compared to subject S1 (Fig. 3A). In this configuration, S2’s stimulation contact was positioned near the intervertebral disc while remaining in the vertebral segment. Representative traces for recorded ESRs and intramuscular EMG recordings at motor threshold, 6.0 mA, were plotted for visual comparisons (Figs. 4B, 4C). RMS was calculated for ECAP and EMG windows of recorded ESRs and intramuscular EMG (IM, time window: 3-13 ms) recordings in evaluating changes in evoked responses for ‘off-disc’ and ‘on-disc’ stimulations. Similar ESRs recorded from S1 (Fig. 2B, red trace), S2’s evoked responses within the ECAP window were significantly larger during ‘on-disc’ stimulation compared to ‘off-disc’ stimulation (Figs. 3B1, 4B). At motor threshold, there were no observable differences within the median traces for motor-related activity in the EMG window of recorded ESRs and intramuscular EMG (Fig. 4B, 4C). Interestingly, although no significant difference was measured within the EMG component of the recorded ESRs, significantly larger evoked motor activity was observed for ‘on-disc’ stimulation within the intramuscular EMG recordings (Figs. 4C, 3B3). The median traces represented within the intramuscular EMG recordings showed no observable differences. However, RMS calculations from individual trials for intramuscular EMG recordings demonstrate that larger responses were evoked throughout the recording (Fig. 4D).

**Fig. 5:**
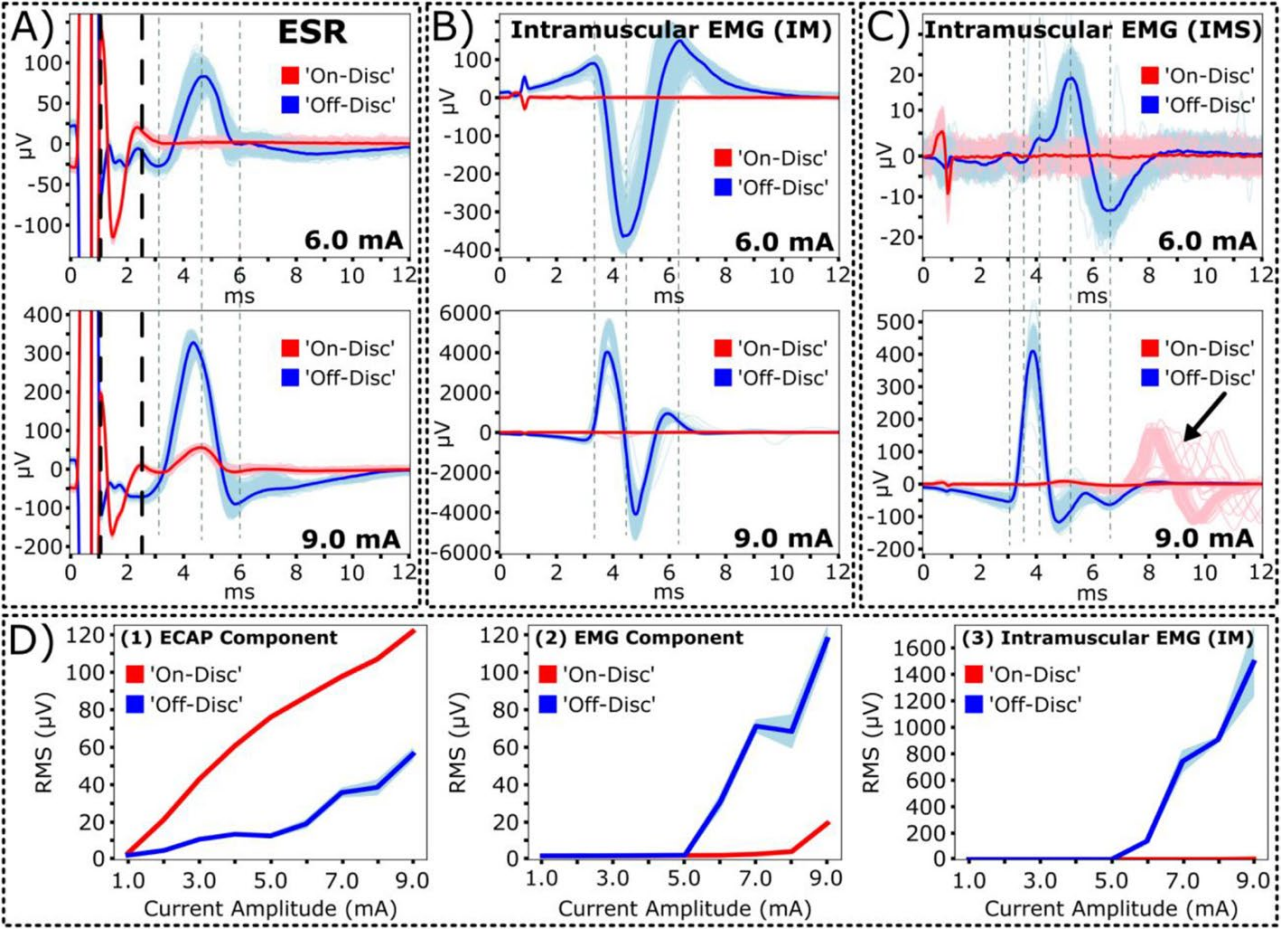
Evoked ESRs are responsive to the anatomical location of the stimulating cathode. Representative data from subject S3. Waveforms were represented as 300 individual recorded traces with an overlaid median trace. **A** Cathodic ‘off-disc’ stimulation resulted in observable EMG bleed-through for recorded ESRs compared to the ‘on-disc’ stimulation. **B** Intramuscular EMG recordings of the intercostal muscles via surgical placement of needle electrodes through the intercostal muscles (IM) and **C** through the skin (IMS) show preference for evoked muscle contraction when cathode was located ‘on-disc’. Cathodic ‘on-disc’ stimulation produced a delayed motor response at the maximum stimulation amplitude, 9.0 mA, for intramuscular EMG (IMS) recordings, as indicated by a black arrow (bottom). **D** Dose-response curves (calculated as root-mean-square, RMS) for collected ESRs, shown as (1) ECAP and (2) EMG components, and (3) intramuscular EMG (IM) recordings show a distinct relationship to cathode placement with respect to the intervertebral disc

We next evaluated recordings from subject S3. Although subject S3’s evoked responses within the ECAP window were consistent with the cohort, trends within the evoked motor-related activity trended in the opposite manner (Fig. 3B2, 3B3). Evoked responses were evaluated at motor thresholds, 6.0 mA, and at the maximum stimulation amplitude for this subject, 9.0 mA. SCS-induced motor responses were observed in the recorded ESRs of representative subject S3 at motor thresholds, 6.0 mA, during 'off-disc' stimulation (Fig. 5A, blue traces). Furthermore, a higher current amplitude, 9.0 mA, was required to elicit motor responses during 'on-disc' stimulation (Fig. 5A, red traces). In evaluating the evoked responses of ‘on-disc’ and ‘off-disc’ stimulation, RMS was calculated for ECAP (time window: 1-2.5 ms) and EMG (time window: 3-13 ms) components of recorded ESRs and intramuscular EMG (time window: 3-13 ms) recordings. Similar to other subjects in the cohort, ‘off-disc’ stimulation produced significantly weaker responses within the ECAP window at the motor threshold, 6.0 mA (-69.1 ± 0.18%; *p*<0.005, paired t-test). and the maximum amplitude 9.0 mA (-42.8 ± 0.18%; *p*<0.005, paired t-test). However, ‘off-disc’ stimulation for subject S3 produced significantly stronger motor activity within the EMG window at the motor threshold, 6.0 mA (1226 ± 1.41%; p<0.005, paired t-test), and the maximum amplitude 9.0 mA (501 ± 3.20%; *p*<0.005, paired t-test). In evaluating the source of this EMG activity, we measured intramuscular EMG activity directly from the intercostal muscles (IM, Fig. 5B) and near the intercostal muscles through inserted needle electrodes in the skin (IMS, Fig. 5C). At the motor threshold (6.0 mA), ‘off-disc’ stimulation produced significantly larger motor activity for both EMG recording sites compared to ‘on-disc’ stimulation (IM: 8550 ± 69.7%; IMS: 372 ± 4.0%; *p*<0.005, paired t-test). At the maximum amplitude, evoked motor activity for ‘off-disc’ stimulation remained significantly larger than ‘on-disc’ stimulation (IM: 33412 ± 951%; IMS: 2404 ± 66.9%; *p*<0.005, paired t-test). As the simulation amplitudes increased from a range of 6.0-9.0 mA, we observed a shift in temporal latency and increased amplitudes of the evoked EMG activity for both intramuscular IM and IMS recordings (Fig. 5B, 5C). No evoked EMG activity was observed directly from intercostal muscles for ‘on-disc’ stimulation (Fig. 5B). However, we observed delayed motor responses that may originate from other muscle groups at 9.0mA when measuring EMG through the skin as opposed to directly from the muscle during ‘on-disc’ stimulation, suggesting motor activation from other muscle groups (Fig. 5C, red traces). RMS quantifications for a time window of 3-13 ms further support that ‘off-disc’ stimulation at current amplitudes above the motor threshold produced delayed motor responses (Supplementary Fig. 4). In evaluating the relationship between stimulation amplitudes for ‘on-disc’ and ‘off-disc’ stimulations, dose-response curves were generated from current amplitudes of 1.0-9.0 mA. ‘On-disc’ stimulation produced significantly larger responses in the ECAP window for all observed stimulation amplitudes compared to ‘off-disc’ stimulation (*p*<0.005, paired t-test; Fig. 5D, first subplot). In addition, for subject S3, ‘off-disc’ stimulation produced significantly larger evoked motor responses at stimulation amplitudes above 5.0 mA, observed in EMG components of recorded ESRs and intramuscular EMG (IM) recordings (*p*<0.005, paired t-test; Fig. 5, D2, D3).

### Sub-centimeter displacements of the stimulation lead can lead to additional motor activation

**Fig. 6:**
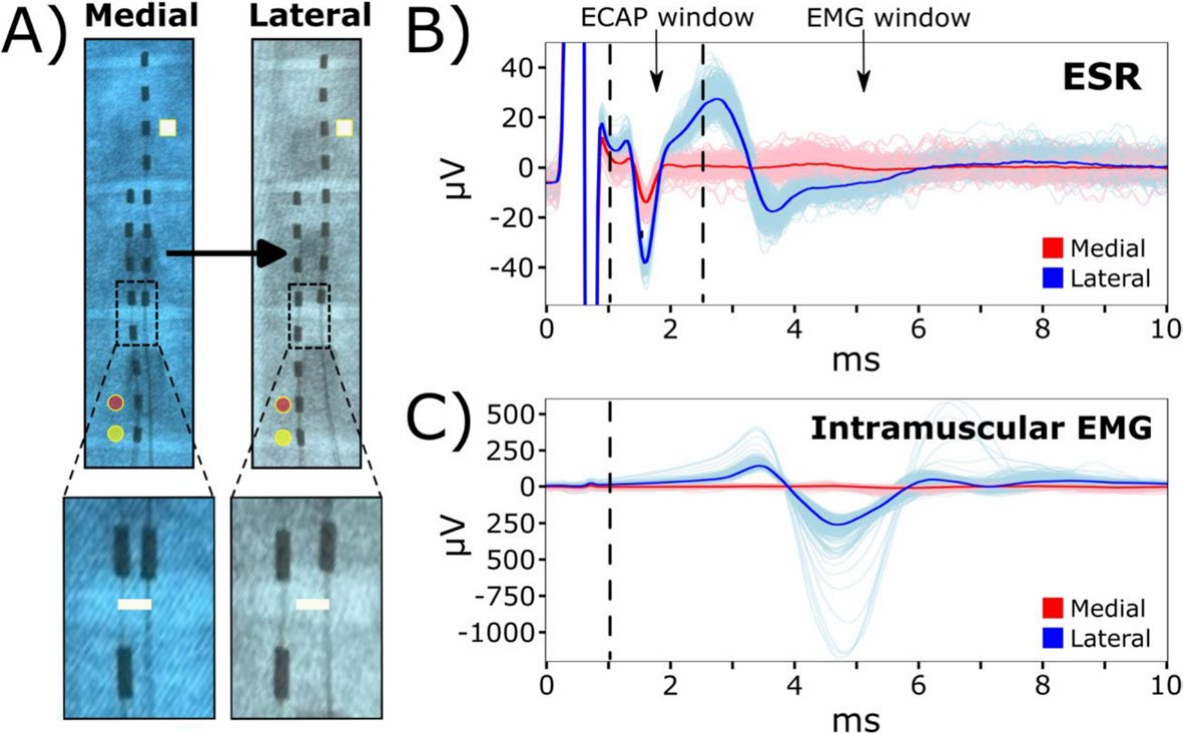
Medio-lateral movement of the stimulation lead may lead to extra muscle recruitment. Representative data from subject S1. **A** Diagram of implanted epidural leads before and after a minimal medial to lateral shift of approximately ~2.9 mm. Contact 11 (white square) on the second lead indicates the recording channel for reported ESR waveforms. Circular markers on the epidural stimulation lead indicate electrode contacts as either anode (yellow) or cathode (red). **B** Recorded ESRs with responses detected in ECAP window (1-2.5 ms) and EMG window (3-13 ms) and, **C** intramuscular EMG (IM) recordings were represented as 300 individual traces with a median trace overlay at motor threshold at the medial lead position, 2.0 mA. EMG window for intramuscular recording is also 3-13 ms. RMS was calculated for ECAP window and EMG window of the recorded ESRs and intramuscular recordings in evaluating changes in evoked responses

Next, we investigate potential changes in evoked ESRs with regard to millimeter displacements of the stimulation lead. The stimulation lead was shifted by hand laterally from the midline at approximately 2.88 ± 0.35 mm for all subjects using fluoroscopic guidance (Fig. 6A, Supplementary Fig. 1). Within representative subject S1, all ESRs included in this analysis had clear evoked signals in ESR and intramuscular EMG (IM) recordings when stimulating at the motor threshold, 2.0 mA, before and after lead displacements (Fig. 6B, 6C). After lead displacement, RMS values of evoked responses within the ECAP window were significantly larger (181 ± 2.72%; *p*<0.005, paired t-test; Fig. 6B). Prior to the lead displacement at medial location, there was no observable EMG component within the recorded ESR traces (Fig. 6B) or EMG responses in intramuscular recording (Fig. 6C). However, after lead displacement, the evoked motor activity was significantly larger in the EMG window of recorded ESRs (326 ± 6.89%; *p*<0.005, paired t-test; Fig. 6B) and intramuscular EMG recordings (1433 ± 54.0%; *p*<0.005, paired t-test; Fig. 6C).

**Fig. 7:**
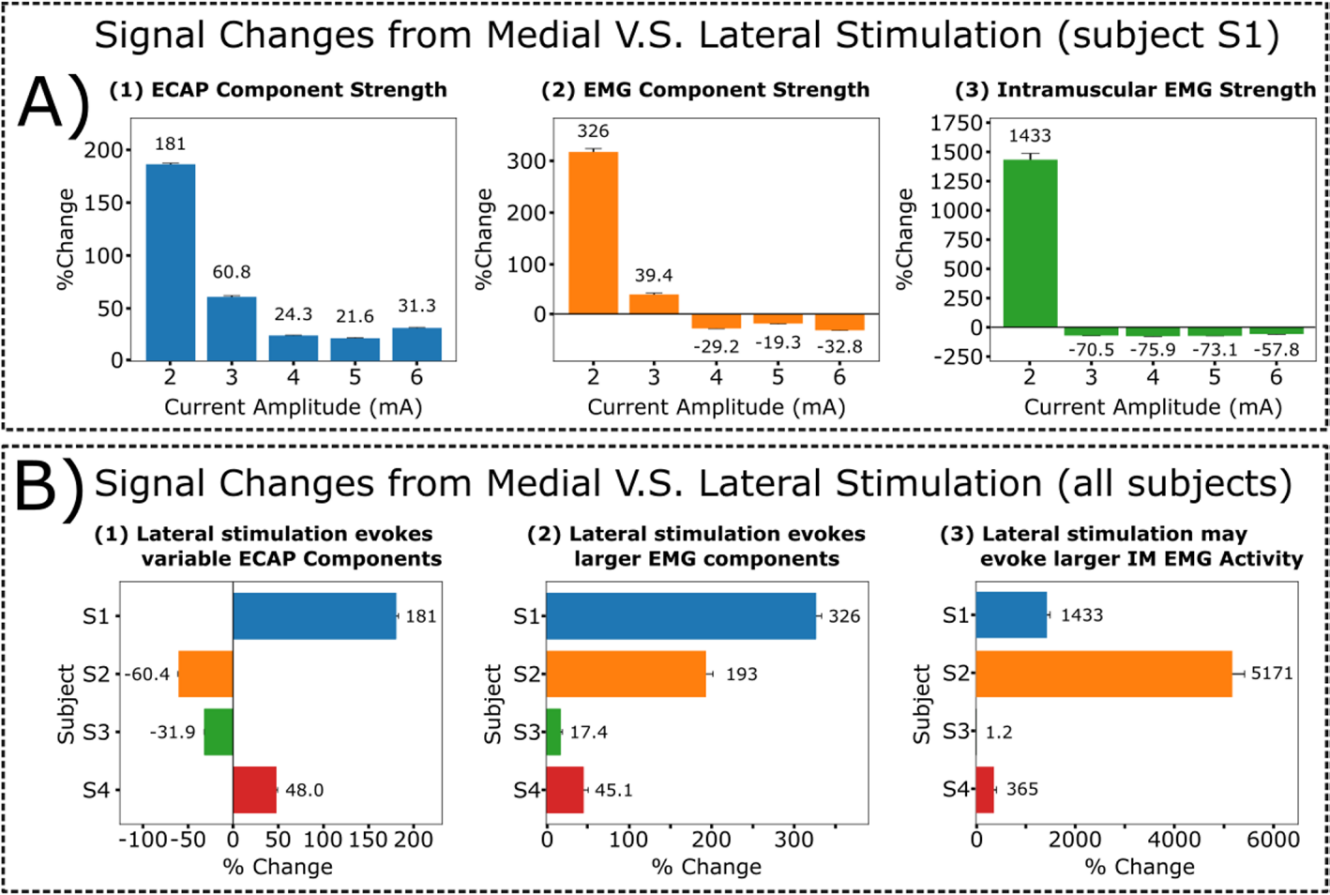
Quantification for recorded ESR components and intramuscular EMG recordings after lateral displacement of the stimulation lead. Motor thresholds for the medial lead position of all subjects (*n*=4) were verified within intramuscular EMG recordings and as follows: S1: 2.0 mA; S2: 6.0 mA; S3: 2.0 mA; S4: 2.9 mA. RMS was calculated for the ECAP (time window: 1-2.5 ms) and EMG (time window: 3-13 ms) components of the recorded ESRs and intramuscular EMG (IM, time window: 3-13 ms). **A** Data from respective subject S1 show signal strength change when stimulation moved from medial to lateral positions across multiple stimulation amplitudes for collected ESRs, shown as (1) ECAP (2.0 mA: 181 ± 2.72%; 3.0 mA: 60.8 ± 1.18%; 4.0 mA: 24.3 ± 0.33%; 5.0 mA: 21.6 ± 0.38%; 6.0 mA: 31.34 ± 0.45%) and (2) EMG components (2.0 mA: 326 ± 6.89%; 3.0 mA: 39.4 ± 2.37%; 4.0 mA: -29.2 ± 0.46%; 5.0 mA: -19.3 ± 0.69%; 6.0 mA: -32.8 ± 0.50%), and (3) intramuscular EMG (IM) recordings (2.0 mA: 1433 ± 54.0%; 3.0 mA: -70.5 ± 0.62%; 4.0 mA: -75.9 ± 0.31%; 5.0 mA: -73.1 ± 0.36%; 6.0 mA: -57.8 ± 0.74%). **B** Changes to signal strength were observed at motor thresholds for all subjects for recorded ESRs, including (1) ECAP (S1: 181 ± 2.72%; S2: -60.4 ± 0.20%; S3: -31.9 ± 0.10%; S4: 48.0 ± 1.20%) and (2) EMG components (S1: 327 ± 6.90%; S2: 193 ± 8.20%; S3: 17.4 ± 2.00%; S4: 45.1 ± 5.50%) and (3) intramuscular EMG (IM) recordings (S1: 1433 ± 54.0%; S2: 5171 ± 237%; S3: 1.20 ± 0.79%; S4: 365 ± 38.9%). Additional plots for subject comparisons containing RMS values are provided in Supplementary Fig. 9

Stimulation at the lateral lead position in subject S1 produced significantly larger responses within the ECAP window across different stimulation amplitudes compared to the medial position (*p*<0.005, paired t-test; Fig. 7A1). Furthermore, stimulation at the lateral lead position produced larger responses within the EMG window of recorded ESRs and intramuscular EMG recordings at the motor threshold, 2.0 mA (*p*<0.005, paired t-test). This relationship declines at higher stimulation amplitudes where the motor-related responses significantly decline at the lateral lead position *p*<0.005, paired t-test, Fig. 7A2, 7A3). In addition, although we observed a significant increase in signal strength within the ECAP component at motor threshold for subject S1 after lateral lead displacement (Fig. 7A1), this trend was not consistent across multiple subjects when stimulating at their respective motor thresholds (Fig. 7B1). Significantly weaker responses for ECAP components after lateral lead displacement were observed for subjects S2 (-60.4 ± 0.20%) and S3 (-31.9 ± 0.10%), while there were significantly stronger responses observed in subjects S1 (181 ± 2.72%) and S4 (48.0 ± 1.20%) (*p*<0.005, paired t-test). In contrast, the changes in motor activity were consistent where stimulation at the lateral lead position led to a significant larger response at respective motor thresholds (Fig. 7, B2, B3), except for subject S3 where a minor increase, 1.2%, was observed in the intramuscular EMG recordings (Fig. 7B3). To investigate the variability among other subjects, a showcase analysis for subject S2 was conducted and a larger voltage fluctuation was detected in the ECAP window when stimulation was located at a more medial location (Supplementary Fig. 5B, Supplementary Fig. 6), which was opposite to what was observed in subject S1 (Fig. 6B). For motor responses, the change was similar when comparing subject S1 to S2, where the lateral stimulation caused a much stronger response in both the EMG component in the ESR and the intramuscular EMG recordings (Supplementary Figs. 5B, 5C, 6). Interestingly, at a lateral position when stimulation is around motor threshold, we observed a bimodal distribution of the individual intramuscular needle recordings (Fig. 6C, Supplementary Fig. 7, at 4.0 mA). Further analysis of the individual recordings along the temporal axis for subject S2 indicated the different muscle recruitments happened at the motor threshold stimulation (Supplementary Fig. 7, at 4.0 mA), and muscle recruitments were unified as the stimulation amplitude was increased (Supplementary Fig. 7, at 6.0 mA and 9 mA).

## Discussion

This case study examines the impact of the micro-neuroanatomy of the spinal cord on optimal stimulation targeting and evoked responses during SCS.

### The role of dorsal roots in SCS-evoked responses

High resolution microCT imaging shows that the arrangement of dorsal and ventral rootlets in the swine's spinal cord is much denser than what is depicted in traditional spinal cord illustrations. In contrast to the significant gaps between the roots and the spinal cord trunk in these illustrations, the rootlets are tightly packed on the surface of the spinal cord (Fig. 1D, Supplementary Fig. 2B). These findings align with an earlier gross cadaver study (Mendez et al. 2021). In this study, reconstructed microCT images revealed that rootlets had diameters as small as 150 µm individually and 1 mm when bundled (Supplementary Fig. 2B, 2C). In comparison, electrode contacts of epidural leads were substantially larger, with a diameter of 1.5 mm and a length of 3 mm (Fig. 1D, Supplementary Fig. 2B).

The DREZ is located in the midline of the spinal cord and consists of a bundle of rootlets. Our microCT data shows that it measures the left DREZ length to be 11.23 mm and right DREZ length to be 15.29 mm. The difference observed between the right and left DREZ might be due to difference at each spinal level specific to an individual animal. However, these results appear to be consistent with a previous study where DREZ (indicated as ‘rostral root–caudal root length’) was about ~10-20 mm (*n*=6) for the lumbar segments (Cuellar et al. 2017). In addition, our results shows that the inter-DREZ distance ranging from 3.05-3.63 mm (Fig. 1D, Supplementary Fig. 2B). This suggests that the stimulation leads may be positioned adjacently or over the dorsal rootlets, where there is likelihood of activating numerous dorsal rootlets during SCS applications. The microCT imaging data showed that there was minimal separation between the dorsal and ventral rootlets where the spinal nerves enter the dura and distribute into rootlets tightly on the surface of the spinal cord. This suggests that ventral rootlets may be within range of the generated electric field during SCS (Supplementary Fig. 2B). Overall, our data suggests the possible activations of both dorsal and ventral rootlets that may lead to direct and/or indirect activation of the motor pathway, leading to unintentional activation of the back muscles. In addition, the evoked motor responses in ESRs and intramuscular EMG recordings, observed at higher stimulation amplitudes, can range from 2-10 ms after the stimulation, further supporting this hypothesis (Figs. 2B, 2C, 3A, 3B, 3C, Supplementary Figs. 5, 6, 7). This might be clinically meaningful to guide lead placement as stimulation leads could be placed more laterally and not aligned with the typical midline placement of the spinal cord.

Electrical stimulation along the rostro-caudal axis, primarily at the intervertebral segment, impacts SCS-evoked responses.

The impact of the stimulation cathode’s location along the rostro-caudal axis was evaluated within each subject individually by reversing the polarity of the anode and cathode, containing an inter-electrode distance of 7 mm. When the cathode was positioned in alignment with the intervertebral disc projection, based on x-ray imaging (termed 'on-disc' stimulation), a significantly weaker response was evoked within the ECAP component of recorded ESRs compared to cases where the cathode was placed away from the disc (termed 'off-disc' stimulation, Figs. 2B, 3A1, 3B1, 4B). Furthermore, ‘on-disc’ stimulation may trigger larger evoked motor-related responses in the EMG component of ESRs and intramuscular EMG recordings (Figs. 2B, 3A2, 3A3). The variability observed in evoked motor responses may be due to the electrode’s position, where ‘on-disc’ stimulation may generate an electrical field that covers more dorsal rootlet distribution compared to fields generated by ‘off-disc’ stimulation (Fig. 1D). As a result, the activation of dorsal rootlets may in-turn activate the ventral rootlets through spinal reflexes, which may further lead to evoked motor responses in the ‘on-disc’ stimulation cases. This is consistent with observations in previous studies, where electrical stimulation of dorsal roots has demonstrated targeted activation of motor fibers (Capogrosso et al. 2013, Greiner et al. 2021).

Interestingly, this trend was not consistent across different subjects when stimulating at the subject’s respective motor thresholds. In subject S2, ‘off-disc’ stimulation produced smaller motor responses at different intensities for the EMG component of the ESR (2.4%) and intramuscular EMG recording (35.1%) (Figs. 3B, 4B, 4C). In subject S3, when stimulating at motor thresholds and above, an opposite trend was observed, where ‘off-disc’ stimulation generated larger motor responses (Figs. 3B, 5B, 5C, 5D). The large variability observed in evoked motor responses could be due to inconsistencies of the electrode placement from animal to animal and the anatomical variabilities of the rootlet distribution across different animals. When the most caudal contact was positioned ~4 mm rostrally from the intervertebral disc for subject S2 (Fig. 4A), stimulation away from the disc may have been inefficient in activating dorsal rootlets, leading to smaller motor responses (Fig. 4B, 4C, Supplementary Fig. 1). Furthermore, dorsal rootlet distribution for some subjects, including S3, may be more caudal concerning the intervertebral disc, observed in x-ray images (Fig. 1D). This suggests that ‘off-disc’ stimulation may lead to better coverage above the rootlets. Thus, ‘off-disc’ stimulation may be more effective in triggering motor responses compared to ‘on-disc’ stimulation observed in this configuration (Fig. 5, Supplementary Fig. 1).

Furthermore, we report that ‘off-disc’ stimulation may lead to delayed motor activity in individual subjects, as observed in latter portions of the 300 recorded trials in intramuscular EMG recordings (Fig. 5C, Supplementary Fig. 4). The onset of delayed motor responses may occur due to unintentional movement of the stimulation lead (e.g., during respiration), resulting in electrical stimulation of densely populated regions of rootlets that may lead to unintended motor activation.

This study demonstrates that the variability observed in the recorded ESRs may be attributed to the anatomical differences of rootlets distribution at different vertebrate level, as well as the difference across different subjects, including the distribution of the dorsal rootlets spanning across the dorsal column (Figs. 2, 4, 5). Future investigations in evaluating anatomical differences across the vertebral column will provide critical information for optimizing SCS therapies. It is important to note that, apart from anatomical considerations, other factors may contribute to variations in recorded ESRs, including the laminectomy procedure, structural variations along the spinal segments, and the acute nature of these experiments.

Sub-centimeter lateral displacement of the stimulating lead impacts SCS-evoked responses.

For subject S1, SCS at the lateral position induced a notably larger signal in the ECAP window compared to stimulation leads placed in the medial position (Fig. 6B). One of the reasons, observing the differences of activation in individual subject, could be that lateral stimulation could have much more coverage on dorsal rootlets, as lateral lead placement will be likely cross the DREZ, which ends more laterally on the spinal cord (Cuellar et al. 2017). However, this observation was inconsistent across different subjects when stimulated at their respective motor thresholds (Fig. 7B1). A further analysis of subject S2 demonstrates an opposite response where the medial stimulation triggered larger responses in the ECAP window (Supplementary Figs. 5, 6). Is it possible that the larger responses observed in subject S2 is due to the recording lead being more susceptible to stimulation artifacts. Our previous study demonstrated that specific placement of the reference electrode significantly influences the impact of stimulation artifacts and the characteristics of the recorded ESRs (Verma et al. 2023). In addition, a shorter latency was observed in the ECAP component of recorded ESRs for this subject (the time from the stimulation artifact to the ECAP’s negative peak) (Supplementary Figs. 5B, 6). One explanation for this unique response is that dorsal rootlets may have been activated for subject S2 during medial stimulation. Lateral stimulation of the dorsal column may produce ECAPs with longer latencies as signal transmission may be delayed from the dorsal rootlets to recording contacts.

Evoked motor responses, including the EMG components of recorded ESRs and intramuscular EMG, were observed more often when electrical stimulation was concentrated around the lateral areas of the spinal cord (Fig. 6B, C, Supplementary Figs. 5, 6). In the lateral position, lower stimulation thresholds for evoked motor responses were observed (Fig. 6C, Supplementary Fig. 3). As the stimulation amplitude was increased, both lateral and medial stimulation were successful in triggering motor responses (Fig. 7A2, 7A3). In contrast, the evoked motor responses were consistently amplified after lateral displacements of the stimulation lead (Figs. 6B, 6C, 7A2, 7A3, 7B2, 7B3). This relationship may be explained by larger dorsal rootlet coverage and activation after lateral displacements of the stimulation lead (Fig. 1D, Supplementary Fig. 2C). In addition, the ventral rootlets and dorsal rootlets divide at the lateral edge of the spinal cord (Supplementary Fig. 2B). As a result, electrical stimulation of laterally placed stimulation leads may stimulate ventral rootlets, leading to unintentional activation of motor responses.

Overall, our results suggest that minimal lead movements within several millimeters can substantially impact the ECAP and EMG components in recorded ESRs. This is clinically significant because patients experience an average lead migration distance of 1.2 cm and 1.7 cm in caudal migration and lateral migration, respectively within the first 3 weeks after implantation (Dombovy-Johnson et al. 2022).

### General limitations

In this study, lead movements were simulated through manual displacements of the epidural leads. Although efforts were made to mitigate variations across animals through x-ray guidance (C-arm) before and after manual lead adjustments, some inherent inconsistencies may exist (Supplementary Fig. 1). These inconsistencies in lead displacement may affect the quantification for the recorded ESRs across subjects. Although anatomical variations among animal subjects may contribute to the observed differences in recorded ESRs, previous studies demonstrated similar SCS effects in swine and the human sensorimotor system, suggesting the relevance of the swine translational model (Cuellar et al. 2017; Islamov et al. 2017; Fadeev et al. 2020; Islamov et al. 2020; Islamov et al. 2022; Verma et al. 2023). Anesthesia also impacts the electrophysiology recordings by influencing spinal reflexes. Furthermore, electrical stimulation and electrophysiology recordings were performed acutely and may not reflect tissue fibrosis and scarring that negatively affects the electrochemical behaviors of the electrode observed in chronic measurements. With regards to 3D-model generations, image overlays were dependent on the alignment of the dorsal root ganglia from stitched 3D-volume renderings of the spinal cord and electrode contact positions obtained from post-operative CBCT scans of the intervertebral foramen. Tissue shrinkage (15%-30%), as a result of the staining process, was estimated based on different imaging modalities and was within established ranges reported in prior literature (Stickland 1975; Pelot et al. 2020). Furthermore, resolution limitations in microCT may restrict the visualization of smaller spinal rootlets.

## Conclusion

In this study, we observed substantial variations in recorded ESRs, including the shape, amplitude, and signal quality. The observed variability in ESR components depends on the location of the stimulation electrode relative to the spinal structures. Unintentional motor activation may occur due to changes in electrode position resulting from the activation of dorsal and ventral rootlets. Following our earlier findings, these results suggest that minimal changes in the electrode positions (in mm) may greatly impact the recordings obtained during spinal cord stimulation. It is essential to consider the impact of anatomical structures and electrode placement on the efficacy of neuromodulation therapy, considering reported electrode migration (in cm) after implantation. To improve stimulation protocols and develop novel neural interfaces for future SCS therapies, it is important to understand the stimulation lead’s location considering the local spinal cord microstructure after implantation and how the evoked response changes over time. This understanding will also help in developing stimulation strategies in closed-loop systems, where different evoked response profiles are utilized to proactively adjust the stimulation contacts to ensure certain neuronal structures are activated, meeting the different needs in various SCS applications.

### Supplementary Information

Additional file1: Supplementary Figure 1. Individual x-ray images for respective subjects show stimulation lead displacement from medial to lateral positions. Supplementary Figure 2. The dorsal and ventral rootlets travel in parallel before separating to their respective sides of the spinal cord, as shown in the T14-T15 intervertebral segment. A) CBCT imaging at the lateral plane shows that the two caudal-most electrode contacts (used for electrical stimulation) were positioned around the T14-T15 intervertebral segment. Dashed white horizontal lines indicate intervertebral discs based on x-ray projections. B) At the T14-T15 intervertebral segment, both dorsal and ventral rootlets travel in close proximity and parallel before their eventual divergence, as shown in the reconstructed microCT image of the spinal cord. The stimulating electrode contact pair was observed to cover a considerable area of the spinal rootlets. C) Prior to the DREZ, the dorsal spinal rootlets were observed either as individual rootlets or grouped, superficial to the dorsal column. Supplementary Figure 3. Median traces for recorded ESRs and intramuscular EMG recordings show observable differences in lateral displacement of the stimulation lead. Representative data from subject S2 where the cathode was located on the intervertebral disk. Waveforms were plotted at various amplitudes beginning at the observable ECAP threshold for this animal. After lead displacement, EMG components of the ESR (top) were observed at higher stimulation amplitudes. Recorded intramuscular EMG highlighted significant amplification with respect to the lead displacement. Supplementary Figure 4. Additional investigations of delayed and evoked motor responses for ‘off-disc’ stimulation (red traces) were conducted for intramuscular EMG recordings from inserted needle electrodes through the skin near the intercostal muscles (IMS). Representative data from subject S1. EMG recordings at stimulation amplitudes at 6.0 mA (top, left) and 9.0 mA (bottom, left) were represented as 300 individual traces with a median trace overlay. RMS values for a time window of 3-13 ms (vertical dotted lines) were quantified across the 300 individual traces for each respective stimulation amplitude (right). Supplementary Figure 5. Medio-lateral movement of the stimulation lead may lead to extra muscle recruitment. Representative data from subject S2. A) Diagram of implanted epidural leads before and after a minimal medial to lateral shift of approximately ~3.2 mm. Contact 11 (white square) on the second lead indicates the recording channel for reported ESR waveforms. Circular markers on the epidural stimulation lead indicate contacts as either anode (yellow) or cathode (red). B) Recorded ESRs with responses detected in the EMG window and C) intramuscular EMG (IM) recordings were represented as 300 individual traces with a median trace overlay at a stimulation amplitude of 4.0 mA. Supplementary Figure 6. Additional quantifications were conducted for the EMG components of recorded ESRs across different stimulation amplitudes at medial and lateral electrode positions. Representative data from subject S2. Recorded ESRs at stimulation amplitudes at 4.0 mA (about the motor threshold for the EMG component in the lateral position), 6.0 mA, and 9.0 mA (left) were represented as 300 individual traces with a median trace overlay. RMS values for a time window of 3.5-10 ms (vertical dotted lines) were quantified across the 300 individual traces for each respective stimulation amplitude (right). Supplementary Figure 7. Additional quantifications were conducted for intramuscular EMG recordings collected from inserted needle electrodes through the intercostal muscles (IM) across different stimulation amplitudes at the medial and lateral positions. Representative data from subject S2. Recorded ESRs at representative stimulation amplitudes at 4.0 mA, 6.0 mA, and 9.0 mA (left) were represented as 300 individual traces with a median trace overlay. RMS values for a time window of 3-10 ms (vertical dotted lines) were quantified across the 300 individual traces for each respective stimulation amplitude (right). Supplementary 8. RMS quantification for recorded ESR components and intramuscular EMG recordings for ‘on-disc’ and ‘off-disc’ stimulation. Changes to signal strength were observed at motor thresholds for all subjects within the recorded ESRs, including (A) ECAP and (B) EMG components and (C) intramuscular EMG (IM) recordings. Supplementary Figure 9. RMS quantification for recorded ESR components and intramuscular EMG recordings for medial and lateral stimulation of the spinal cord. Changes to signal strength were observed at motor thresholds for all subjects within the recorded ESRs, including (A) ECAP and (B) EMG components and (C) intramuscular EMG (IM) recordings.

## Data Availability

The data used in this study was fully funded by Abbott and is the proprietary and confidential property of Abbott. Abbott is under no obligation to release the data to third parties.
