# Correction: The role of spinal cord neuroanatomy in the variances of epidural spinal recordings

**DOI:** 10.1186/s42234-024-00152-7

**Published:** 2024-08-07

**Authors:** Danny V. Lam, Justin Chin, Meagan K. Brucker-Hahn, Megan Settell, Ben Romanauski, Nishant Verma, Aniruddha Upadhye, Ashlesha Deshmukh, Aaron Skubal, Yuichiro Nishiyama, Jian Hao, J. Luis Lujan, Simeng Zhang, Bruce Knudsen, Stephan Blanz, Scott F. Lempka, Kip A. Ludwig, Andrew J. Shoffstall, Hyun-Joo Park, Erika Ross Ellison, Mingming Zhang, Igor Lavrov

**Affiliations:** 1 Neural Lab, Abbott Neuromodulation, Plano, TX USA; 2 Department of Biomedical Engineering, Case Western Reserve University, Cleveland, OH USA; 3 Department of Veterans Affairs Medical Center, Advanced Platform Technology Center, Louis Stokes Cleveland, Cleveland, OH USA; 4 Department of Neurosurgery, Mayo Clinic, Rochester, MN USA; 5 Department of Biomedical Engineering, University of Wisconsin Madison, Madison, USA; 6 Wisconsin Institute for Translational Neuroengineering (WITNe), Madison, WI USA; 7 Department of Neurology, Mayo Clinic, Rochester, MN USA; 8 Department of Physiology and Biomedical Engineering, Mayo Clinic, Rochester, MN USA; 9 University of Wisconsin School of Medicine and Public Health, Madison, WI USA; 10 Department of Biomedical Engineering, University of Michigan, Ann Arbor, MI USA; 11 Biointerfaces Institute, University of Michigan, Ann Arbor, MI USA; 12 Department of Anesthesiology, University of Michigan, Ann Arbor, MI USA; 13 Department of Neurosurgery, University of Wisconsin-Madison, Madison, WI USA

**Correction:*Bioelectron Med 10***, **17 (2024)**

10.1186/s42234-024-00149-2

Following publication of the original article (Lam et al. 2024), the authors identified the below errors:

1. The article title ‘The role of spinal cord neuroanatomy and the variances of epidurally evoked spinal responses’ should be corrected to ‘The role of spinal cord neuroanatomy **in the** variances of epidural spinal **recordings**’.
2. In ‘Conclusion’, the last sentence was cut off. The missing data was highlighted in bold below.

Incorrect sentence: This understanding will also help in developing stimulation strategies in closed-loop systems, where different evoked response profiles are utilized to proactively adjust the stimulation contacts to ensure certain neuronal structures are activated, meeting the different

Correct sentence: This understanding will also help in developing stimulation strategies in closed-loop systems, where different evoked response profiles are utilized to proactively adjust the stimulation contacts to ensure certain neuronal structures are activated, meeting the different **needs in various SCS applications**.

3. Additional file 1 was incorrect. The correct version is included in this correction article.

The original article (Lam et al. 2024) has been corrected.

### Electronic supplementary material

Below is the link to the electronic supplementary material.

Supplementary Material 1

## References

[CR1] Lam DV, Chin J, Brucker-Hahn MK, et al. The role of spinal cord neuroanatomy and the variances of epidurally evoked spinal responses. Bioelectron Med. 2024;10:17. 10.1186/s42234-024-00149-2. 39020366 10.1186/s42234-024-00149-2 PMC11253499

