# Supplementary material for: The role of spinal cord neuroanatomy in the variances of epidural spinal recordings
Source: Bioelectron Med. 2024 Jul 17;10:17. doi: 10.1186/s42234-024-00149-2 (PMC11253499; doi:10.1186/s42234-024-00149-2)
Supplement: Supplementary file 1 — Additional file1: Supplementary Figure 1. Individual x-ray images for respective subjects show stimulation lead displacement from medial to lateral positions. Supplementary Figure 2. The dorsal and ventral rootlets travel in parallel before separating to their respective sides of the spinal cord, as shown in the T14-T15 intervertebral segment. A) CBCT imaging at the lateral plane shows that the two caudal-most electrode contacts (used for electrical stimulation) were positioned around the T14-T15 intervertebral segment. Dashed white horizontal lines indicate intervertebral discs based on x-ray projections. B) At the T14-T15 intervertebral segment, both dorsal and ventral rootlets travel in close proximity and parallel before their eventual divergence, as shown in the reconstructed microCT image of the spinal cord. The stimulating electrode contact pair was observed to cover a considerable area of the spinal rootlets. C) Prior to the DREZ, the dorsal spinal rootlets were observed either as individual rootlets or grouped, superficial to the dorsal column. Supplementary Figure 3. Median traces for recorded ESRs and intramuscular EMG recordings show observable differences in lateral displacement of the stimulation lead. Representative data from subject S2 where the cathode was located on the intervertebral disk. Waveforms were plotted at various amplitudes beginning at the observable ECAP threshold for this animal. After lead displacement, EMG components of the ESR (top) were observed at higher stimulation amplitudes. Recorded intramuscular EMG highlighted significant amplification with respect to the lead displacement. Supplementary Figure 4. Additional investigations of delayed and evoked motor responses for ‘off-disc’ stimulation (red traces) were conducted for intramuscular EMG recordings from inserted needle electrodes through the skin near the intercostal muscles (IMS). Representative data from subject S1. EMG recordings at stimulation amplitudes at 6.0 mA (top, l [file 42234_2024_149_MOESM1_ESM.docx]

Supplementary Materials


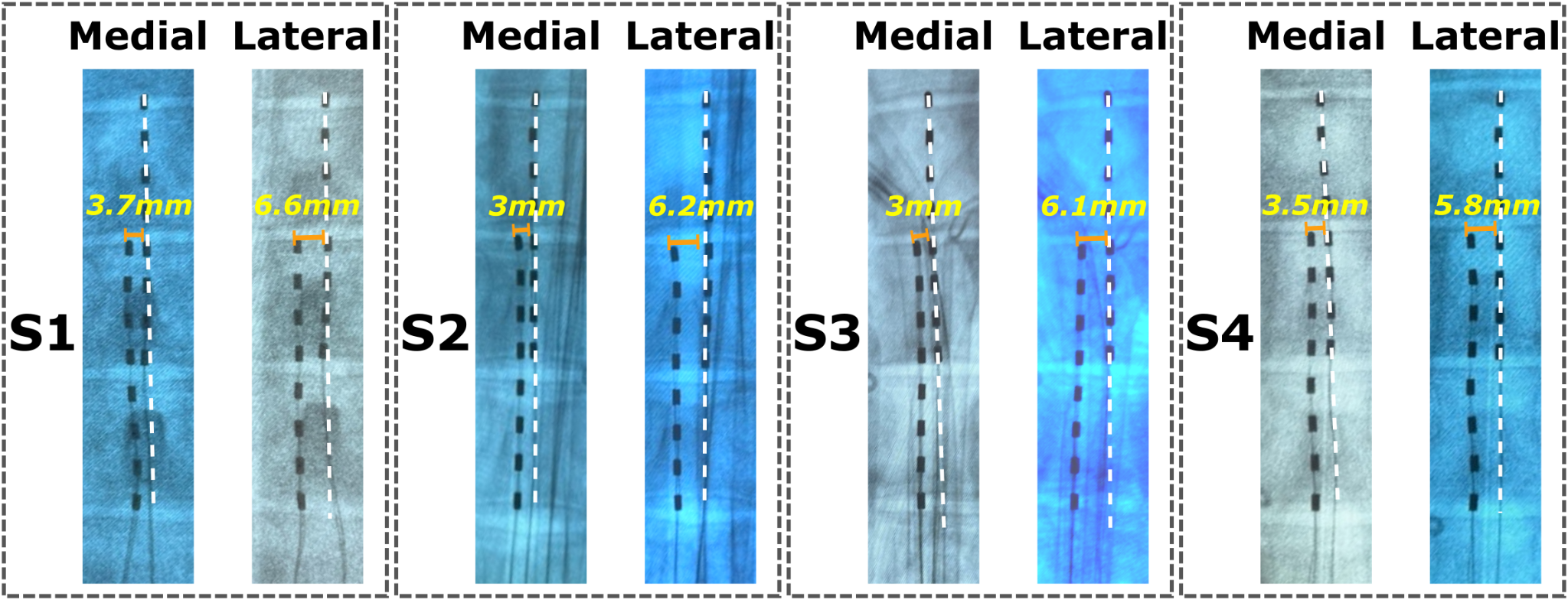


**Supplementary Figure 1.** Individual x-ray images for respective subjects show stimulation lead displacement from medial to lateral positions.


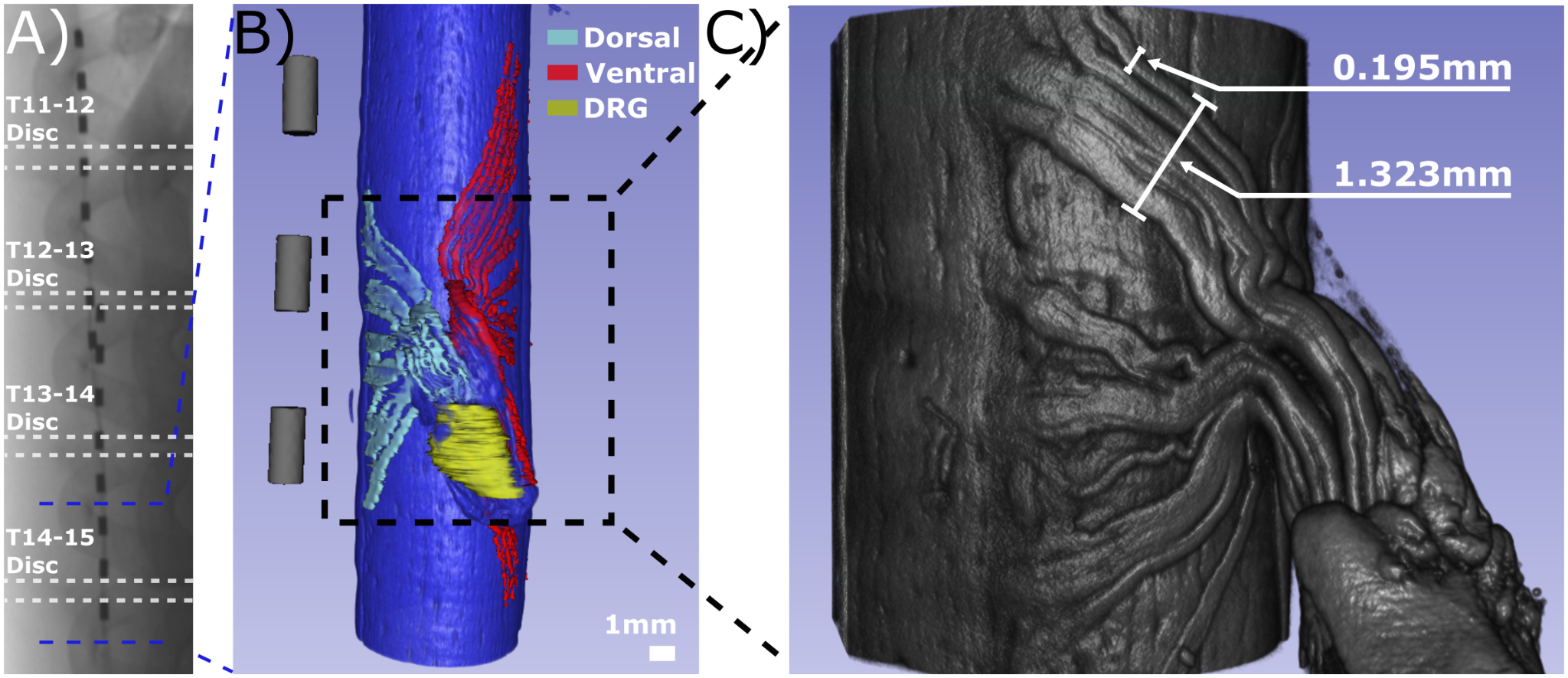


**Supplementary Figure 2.** The dorsal and ventral rootlets travel in parallel before separating to their respective sides of the spinal cord, as shown in the T14-T15 intervertebral segment. A) CBCT imaging at the lateral plane shows that the two caudal-most electrode contacts (used for electrical stimulation) were positioned around the T14-T15 intervertebral segment. Dashed white horizontal lines indicate intervertebral discs based on x-ray projections. B) At the T14-T15 intervertebral segment, both dorsal and ventral rootlets travel in close proximity and parallel before their eventual divergence, as shown in the reconstructed microCT image of the spinal cord. The stimulating electrode contact pair was observed to cover a considerable area of the spinal rootlets. C) Prior to the DREZ, the dorsal spinal rootlets were observed either as individual rootlets or grouped, superficial to the dorsal column.


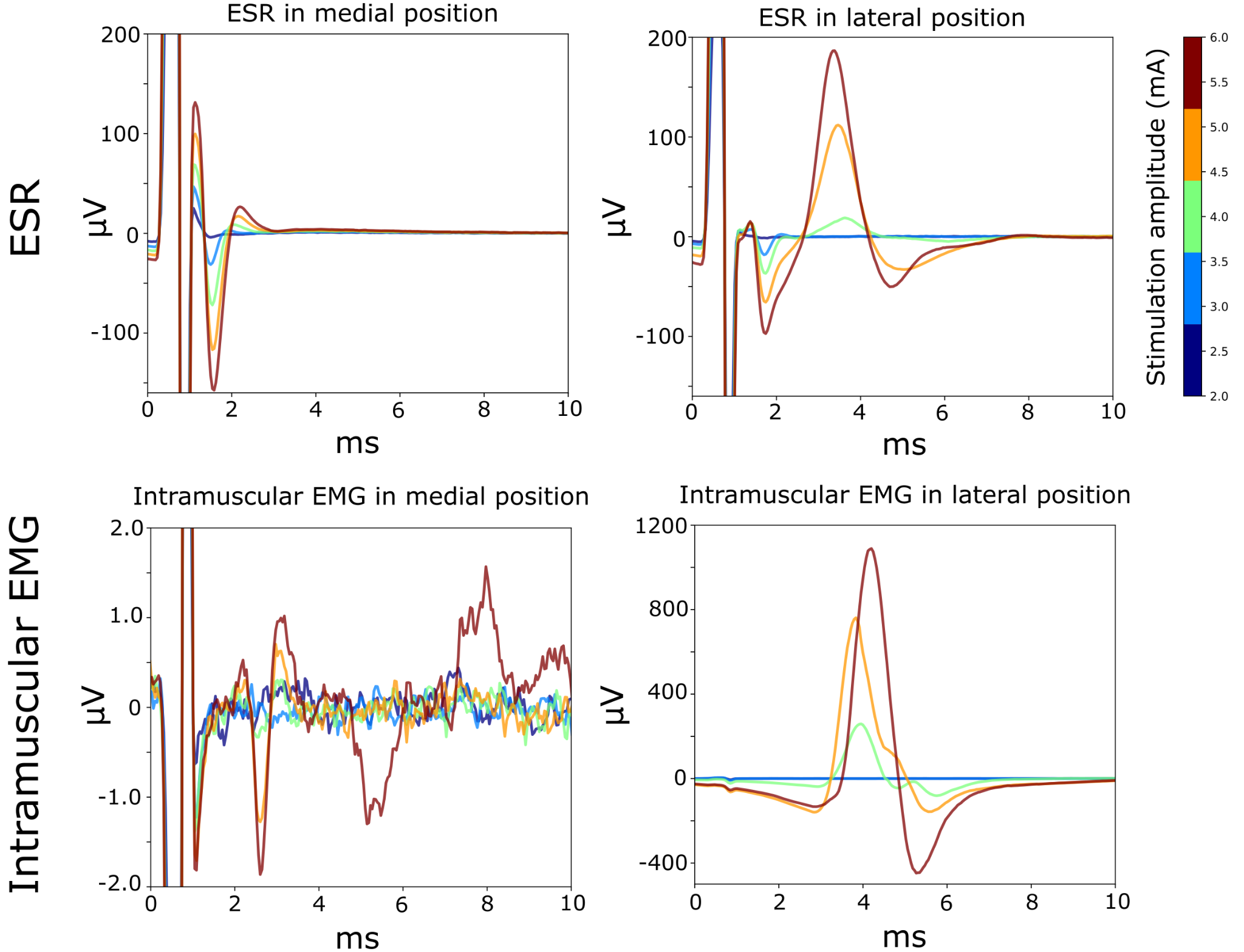


**Supplementary Figure 3.** **Median traces for recorded ESRs and intramuscular EMG recordings show observable differences in lateral displacement of the stimulation lead.** Representative data from subject S2 where the cathode was located on the intervertebral disk. Waveforms were plotted at various amplitudes beginning at the observable ECAP threshold for this animal. After lead displacement, EMG components of the ESR (top) were observed at higher stimulation amplitudes. Recorded intramuscular EMG highlighted significant amplification with respect to the lead displacement.


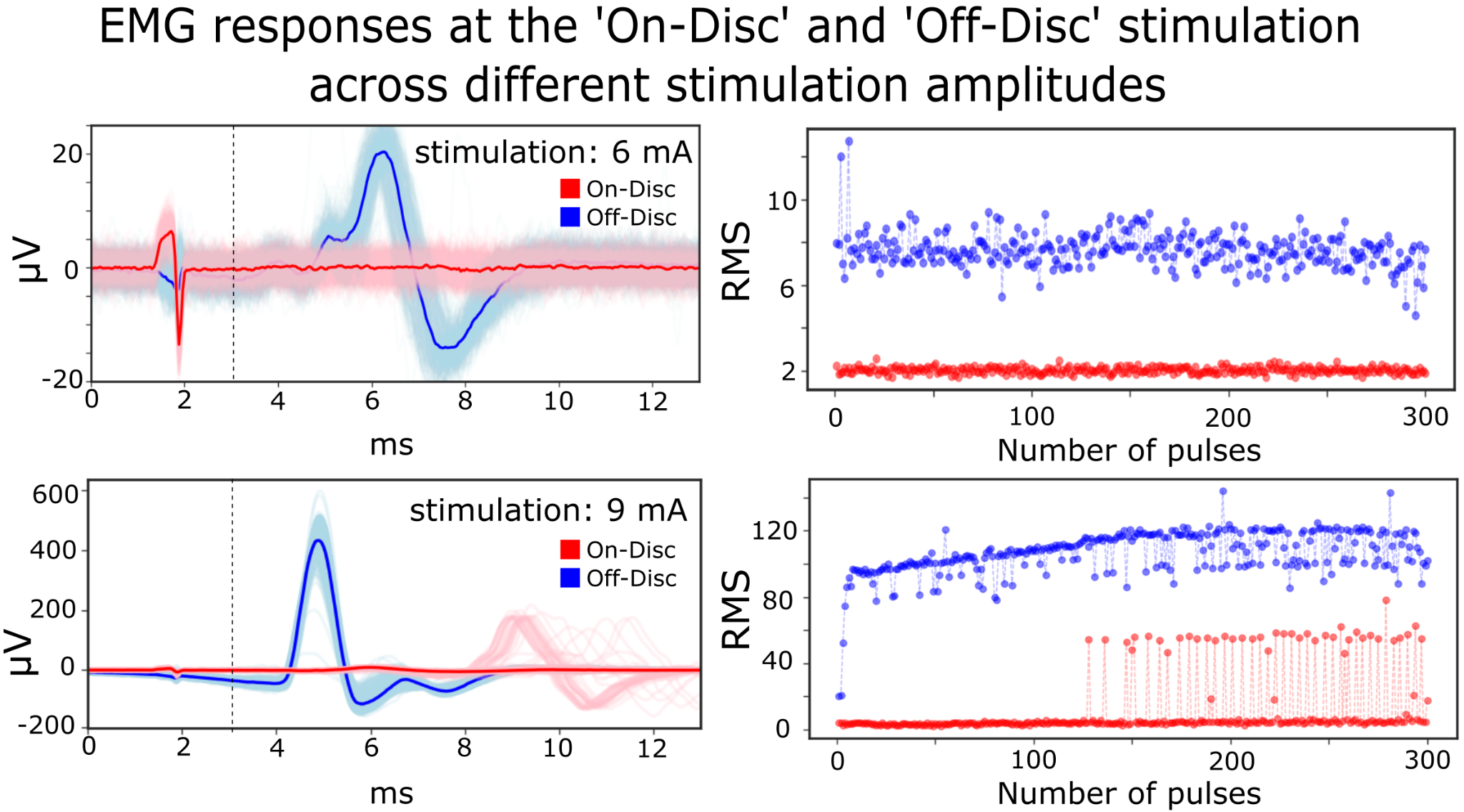


**Supplementary Figure 4**. **Additional investigations of delayed and evoked motor responses for ‘off-disc’ stimulation (red traces) were conducted for intramuscular EMG recordings from inserted needle electrodes through the skin near the intercostal muscles (IMS).** Representative data from subject S1. EMG recordings at stimulation amplitudes at 6.0 mA (top, left) and 9.0 mA (bottom, left) were represented as 300 individual traces with a median trace overlay. RMS values for a time window of 3-13 ms (vertical dotted lines) were quantified across the 300 individual traces for each respective stimulation amplitude (right).


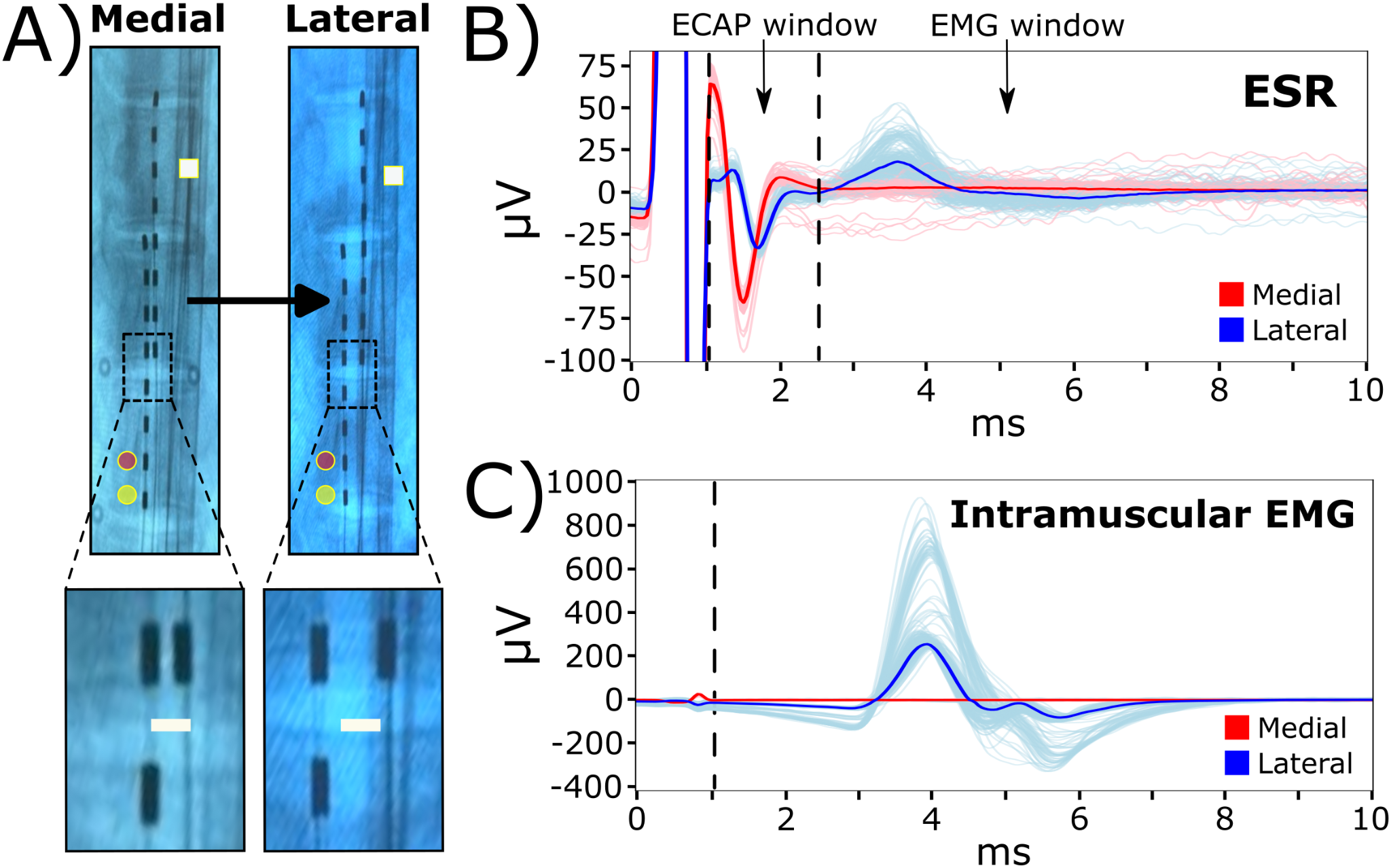


**Supplementary Figure 5. Medio-lateral movement of the stimulation lead may lead to extra muscle recruitment**. Representative data from subject S2. A) Diagram of implanted epidural leads before and after a minimal medial to lateral shift of approximately ~3.2 mm. Contact 11 (white square) on the second lead indicates the recording channel for reported ESR waveforms. Circular markers on the epidural stimulation lead indicate contacts as either anode (yellow) or cathode (red). B) Recorded ESRs with responses detected in the EMG window and C) intramuscular EMG (IM) recordings were represented as 300 individual traces with a median trace overlay at a stimulation amplitude of 4.0 mA


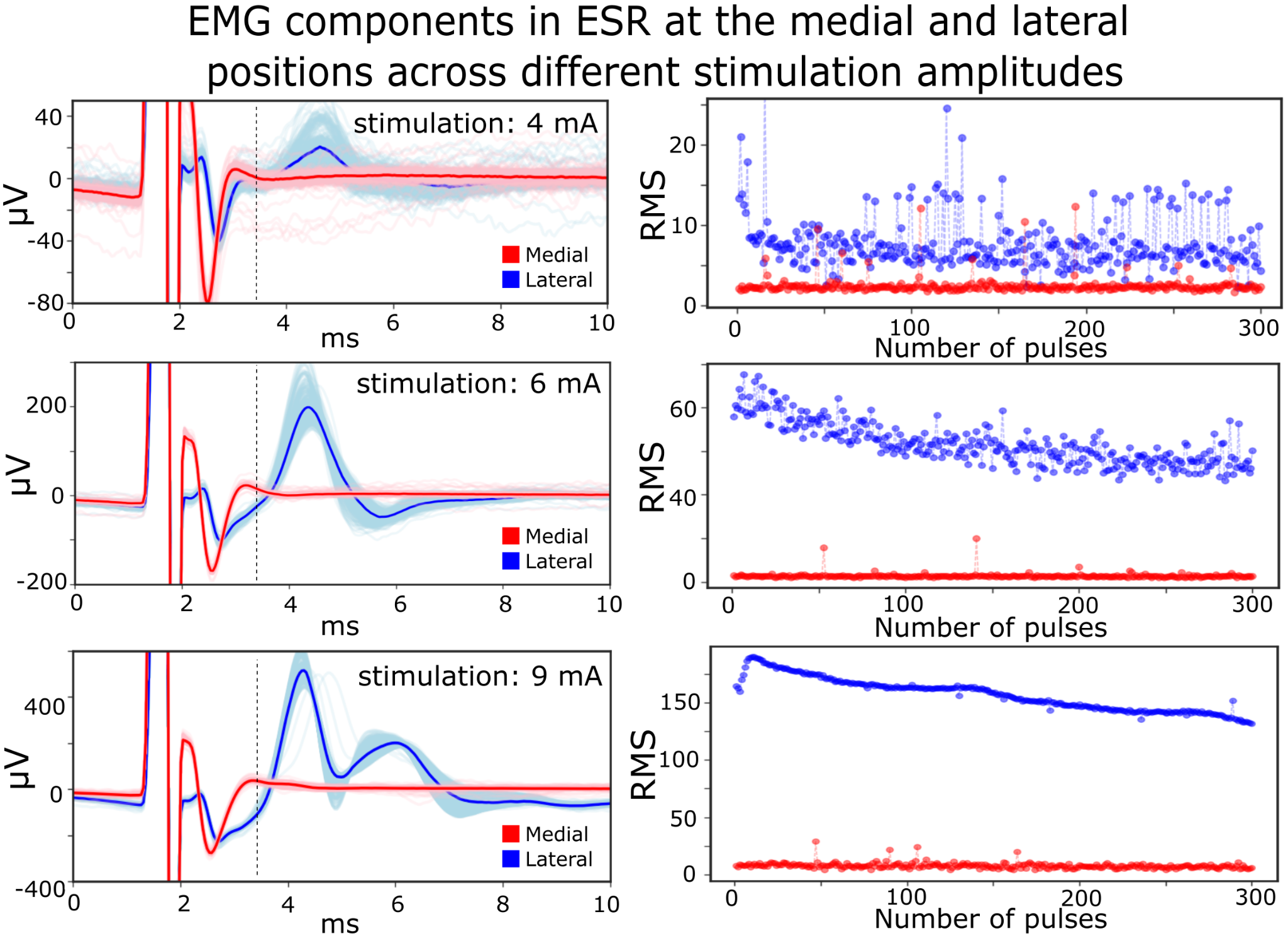


**Supplementary Figure 6**. **Additional quantifications were conducted for the EMG components of recorded ESRs across different stimulation amplitudes at medial and lateral electrode positions.** Representative data from subject S2**.** Recorded ESRs at stimulation amplitudes at 4.0 mA (about the motor threshold for the EMG component in the lateral position), 6.0 mA, and 9.0 mA (left) were represented as 300 individual traces with a median trace overlay. RMS values for a time window of 3.5-10 ms (vertical dotted lines) were quantified across the 300 individual traces for each respective stimulation amplitude (right).


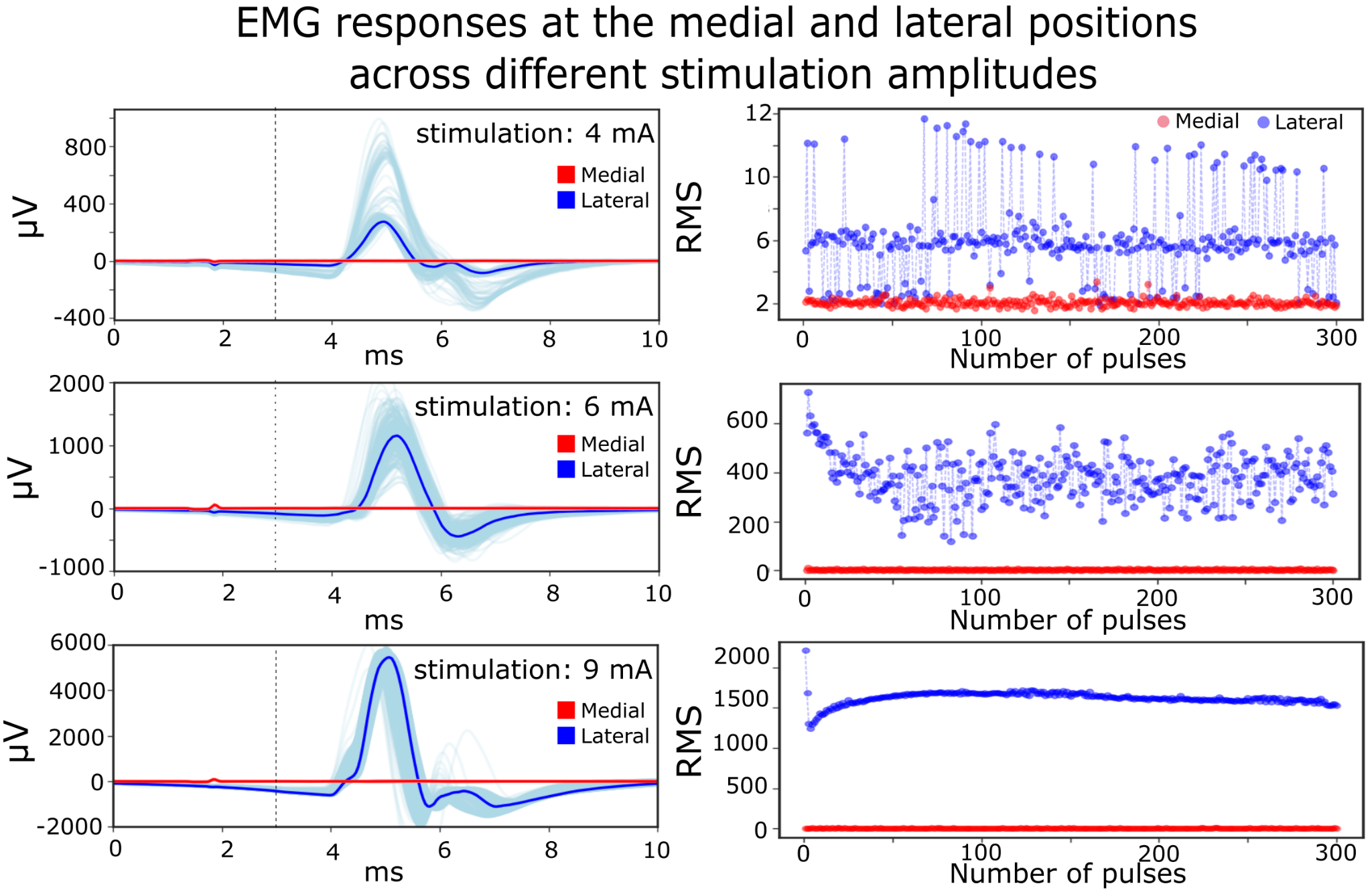


**Supplementary Figure 7. Additional quantifications were conducted for intramuscular EMG recordings collected from inserted needle electrodes through the intercostal muscles (IM) across different stimulation amplitudes at the medial and lateral positions.** Representative data from subject S2. Recorded ESRs at representative stimulation amplitudes at 4.0 mA, 6.0 mA, and 9.0 mA (left) were represented as 300 individual traces with a median trace overlay. RMS values for a time window of 3-10 ms (vertical dotted lines) were quantified across the 300 individual traces for each respective stimulation amplitude (right).


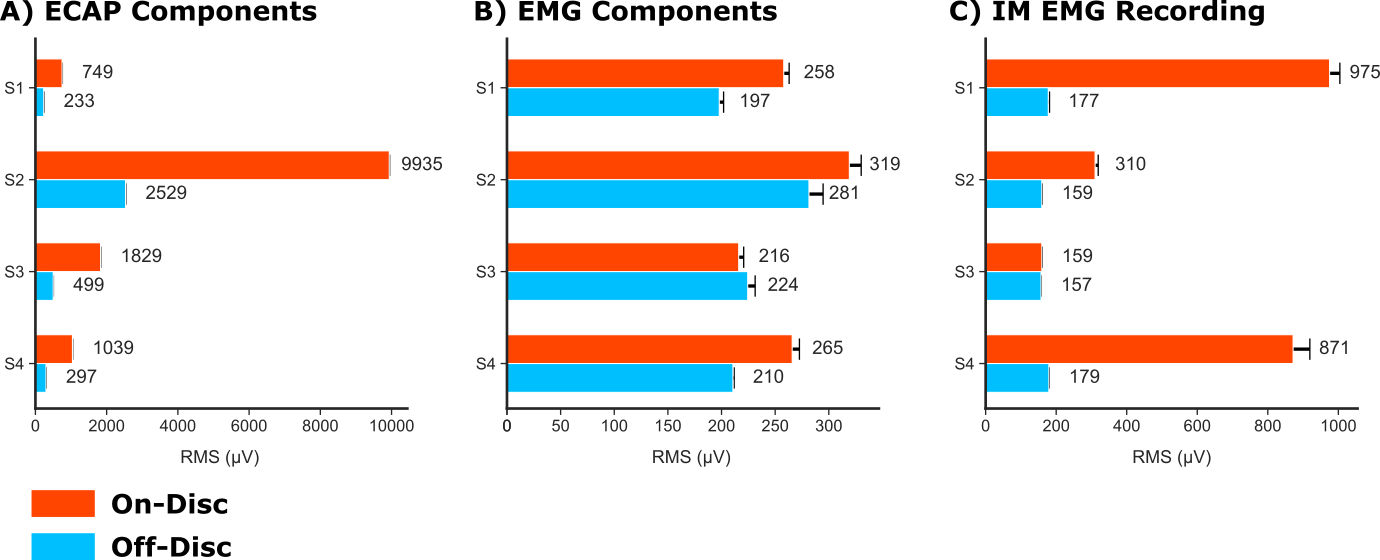


**Supplementary Figure 8. RMS quantification for recorded ESR components and intramuscular EMG recordings for ‘on-disc’ and ‘off-disc’ stimulation.** Changes to signal strength were observed at motor thresholds for all subjects within the recorded ESRs, including (A) ECAP and (B) EMG components and (C) intramuscular EMG (IM) recordings.


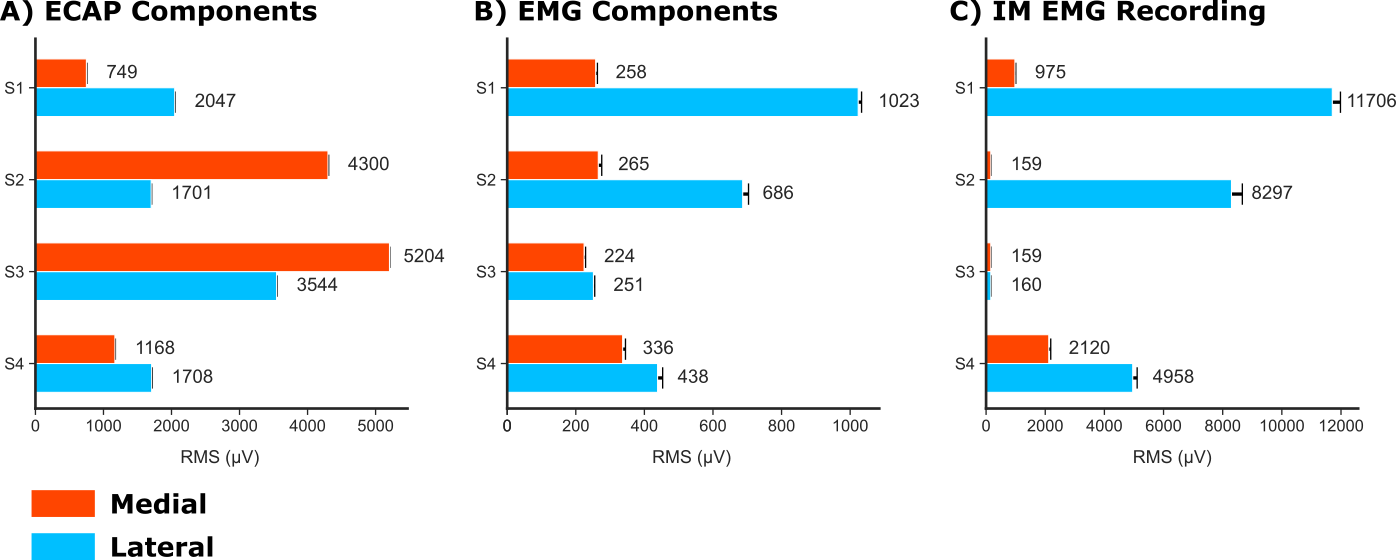


**Supplementary Figure 9. RMS quantification for recorded ESR components and intramuscular EMG recordings for medial and lateral stimulation of the spinal cord.** Changes to signal strength were observed at motor thresholds for all subjects within the recorded ESRs, including (A) ECAP and (B) EMG components and (C) intramuscular EMG (IM) recordings.
